# Oriented suspension mechanics with application to improving flow linear dichroism spectroscopy

**DOI:** 10.1098/rspa.2019.0184

**Published:** 2019-12-18

**Authors:** G. Cupples, D. J. Smith, M. R. Hicks, R. J. Dyson

**Affiliations:** 1 School of Mathematics, University of Birmingham, Birmingham B15 2TT, UK; 2 Linear Diagnostics Ltd, BioHub Birmingham, 97 Vincent Drive, Birmingham B15 2SQ, UK

**Keywords:** flow-induced alignment, linear dichroism spectroscopy, Brownian suspensions, Fokker–Planck equation

## Abstract

Flow linear dichroism is a biophysical spectroscopic technique that exploits the shear-induced alignment of elongated particles in suspension. Motivated by the broad aim of optimizing the sensitivity of this technique, and more specifically by a hand-held synthetic biotechnology prototype for waterborne-pathogen detection, a model of steady and oscillating pressure-driven channel flow and orientation dynamics of a suspension of slender microscopic fibres is developed. The model couples the Fokker–Planck equation for Brownian suspensions with the narrow channel flow equations, the latter modified to incorporate mechanical anisotropy induced by the particles. The linear dichroism signal is estimated through integrating the perpendicular components of the distribution function via an appropriate formula which takes the biaxial nature of the orientation into account. For the specific application of pathogen detection via binding of M13 bacteriophage, it is found that increases in the channel depth are more significant in improving the linear dichroism signal than increases in the channel width. Increasing the channel depth to 2 mm and pressure gradient to 5 × 10^4^ Pa m^−1^ essentially maximizes the alignment. Oscillating flow can produce nearly equal alignment to steady flow at appropriate frequencies, which has significant potential practical value in the analysis of small sample volumes.

## 1. Introduction

Suspensions of particles in liquid or gas are found throughout the natural world and in many industrial processes; including blood, particulate-laden air, algae in open water or in bioconvection experiments, semen samples and industrial applications such as emulsions in food and cosmetics—these examples and more are elaborated in [1–5]. We will consider flowing suspensions of microscopic elongated rod-like particles, as a means to understand and improve flow linear dichroism spectroscopy, with particular focus on a specific technological application. The core idea of flow linear dichroism spectroscopy is that elongated particles undergo shear-induced rotation, which has the effect of concentrating their orientation in the direction of flow. The difference in absorbance of light polarized parallel, and perpendicular to, the flow direction can be used to reveal structural information; further information and wider context is given in [6–9]. The application of mathematical modelling to understand and quantify molecular orientation in flow linear dichroism was addressed by McLachlan *et al.* [10], building on classical oriented suspension mechanics [11,12]. This manuscript will generalize this work to the significantly more complex problem of a non-homogeneous shear environment of pressure-driven, and potentially time-varying, channel flow, and will moreover focus on a specific technological application. The methods and results will be adaptable to linear dichroism spectroscopy and beyond.

The specific application is a prototype hand-held device, developed by Linear Diagnostics Ltd (LD) designed to detect waterborne pathogens in fluids. The analyte is mixed with a reagent containing a synthetic biology micrometre-length fibre based on M13 bacteriophage, a filamentous virus known to infect Gram-negative bacteria (for example, *Escherichia coli*) [13]. M13 easily align in shear flow due to their relative rigidity and large aspect ratio, and are readily genetically engineered [14]. Microscopic elongated particles have been used to measure shear in microfluidic devices [15] and to determine wall shear stress in small biological structures [16]. In the absence of the pathogen, the fibres align in the flow field and therefore exhibit linear dichroism. The M13 are engineered with antibodies enabling binding to specific pathogens, therefore in the presence of the pathogen the alignment is disrupted, resulting in a reduced linear dichroism signal. The technique is described further in [14]. To improve the sensitivity of the device it is desirable to optimize the signal to noise ratio, which can be achieved by increasing the overall alignment in the system. To analyse the system theoretically involves solving a coupled pressure-driven steady or oscillating flow and alignment problem. While we will present results for a specific biotechnology application, the computational framework is generic to any channel flow of dilute suspensions.

The coupling between the flow and particle orientation is important to understand the dynamics of these suspensions. Jeffery [17] carried out a landmark study of the dynamics of suspended elongated particles in viscous flow, in particular, shear-induced alignment. Moreover, following seminal theoretical work [18–21], it has been established how the aspect ratio of particles affects emergent rheology. In the related context of suspensions of actively-swimming oriented particles, Pedley & Kessler [22] described a coupled model of orientation distribution, governed by a Fokker–Planck equation, and fluid flow, given by modifications to the Navier–Stokes equations proportional to the ensemble averages of orientation. These early studies form the theoretical framework for our model of homogeneous suspensions of semi-rigid M13 bacteriophage via the approximation of elongated, axisymmetric, rigid Brownian rods in the limit of infinite aspect ratio.

Other relevant theoretical analyses include work on homogeneous shear [12,23,24], time-dependent shear [25], turbulent channel flow [26–28] and pressure-driven channel flow [29]. Including variations in particle concentration in channels has been investigated in a weak flow limit [30] and when the particle size is comparable to the channel width [31].

More recently, Taylor–Couette [32] and Rayleigh–Bénard [33] stability of perfectly aligned suspensions have been theoretically modelled via the transversely isotropic fluid of Ericksen [18,34]. Similar models have also been used to investigate fibrous biological systems such as growth in plant cell walls [35], propulsion in aligned cervical mucus [36,37] and the extracellular matrix [38].

This paper solves and compares two three-dimensional (3D) pressure-driven flow problems for an oriented suspension in a narrow rectangular channel geometry: steady flow, which models the continuously pumped loop used in existing spectroscopic devices, including the LD technology, and a potential novel oscillatory system, in which a smaller volume of analyte is oscillated back and forth. For practical particle concentrations, we have n*_d_a*^3^ < 1, where n*_d_ is particle number density and a* is the particle length, and hence the suspension is at the upper end of the *dilute* range [39]; this theory has previously been shown to hold for n*_d_a*^3^ ≈ 1 [30]. The channel dimensions, pressure gradient, particle number density and frequency of oscillations are investigated as factors to improve alignment, and thus signal, in both flow types and to determine the viability of an oscillatory system for aligning particles.

The coupled orientation and flow model will be solved numerically by iterative coupling. Mathematical modelling of these suspensions is computationally challenging due to the additional independent variables associated with the particle degrees of freedom and the coupling between particle dynamics, velocity gradients and rheology. Rational simplification of the flow problem via lubrication theory, the application of a spectral method [12] and multicore parallelization of the array of spatially local orientation problems, will be shown to enable practical solution with workstation hardware.

The manuscript is organized as follows: the governing equations for the system, including the Navier–Stokes and Fokker–Planck equations, are summarized in §2. The steady flow model is presented in §3 and the oscillatory problem in §4. Results for both flow problems are presented in §5 and discussed and compared in §6.

## 2. Summary of equations governing dilute suspensions of elongated particles

**Figure 1.**
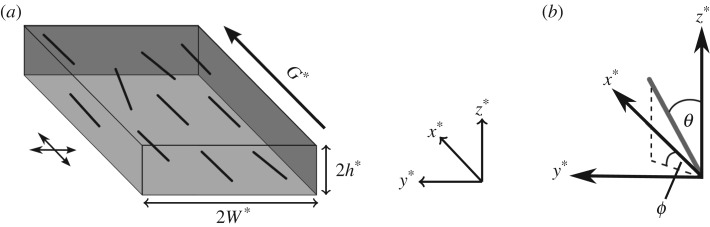
Schematic of the flow of a fibre-laden fluid, forced by pressure gradient G* with a distribution of orientation, in a thin rectangular channel. (*a*) Rectangular channel of width 2W* and depth 2h* and the x*-axis is the molecular orientation axis. The orientation parameter is calculated as the proportional difference in orientation parallel and perpendicular to the molecular orientation axis, highlighted by the arrows to the left of the channel. Note that for oscillating flow, the pressure gradient is replaced with G*e^iω*t*^ for frequency of oscillations ω*. (*b*) The orientation angles *θ*∈[0, *π*] and *ϕ*∈[0, 2*π*) away from the coordinate axis describe the orientation of the fibres.

Consider a 3D rectangular channel of width 2W*, depth 2h* and length-scale L*, where h*, W*≪L*. The axis origin is at the centre of the channel and it is assumed that the flow direction induced by pressure gradient G*, or the molecular orientation axis, is the x*-direction (figure 1*a*). The behaviour of dilute suspensions is approximated by altering the constitutive equation for the stress tensor σ* [40,41]; the motivating problem is at the upper end of the dilute regime (see §5). This constitutive equation, along with the coupled Fokker–Planck equation governing fibre behaviour [12], are briefly detailed below.

The dimensional Navier–Stokes and incompressibility equations are

2.1 $$\rho^{*}\left(\frac{\partial u^{*}}{\partial t^{*}}+(u^{*}\cdot \nabla^{*})u^{*}\right)=\nabla^{*}\cdot \sigma^{*}$$

and

2.2 $$\nabla^{*}\cdot u^{*}=0,$$

where ρ* is density, u*(x*, t*) is the velocity vector for spatial coordinate x* and time t* and ∇* is differentiation in positional coordinates. The asterisk notation represents dimensional variables. The boundary conditions are given by no-slip and no-penetration at the walls,

2.3 $$u^{*}(x^{*},\pm W^{*},z^{*},t^{*})=0\;\mathrm{and}\;u^{*}(x^{*},y^{*},\pm h^{*},t^{*})=0.$$

The boundary conditions for x* have not been defined here as they are specified for each flow system.

To determine the bulk stresses in the fluid, consider a homogeneous suspension of particles, dilute enough to ensure that particle–particle interactions are negligible [10,12]. Nitsche & Hinch [42] found that anisotropic translational diffusivity can produce migration and hence non-uniform concentration. In the present motivating problem, we estimate the timescale for translational diffusion as h^2^/D_T_ ≈ (3 × 10^−4^)^2^/10^−13^ > ∽10^5^ s and hence this effect will not be significant here. Furthermore, assuming the cell density is spatially homogeneous, the suspension configuration can be described by the orientation distribution function *ψ*(x*, **p**, t*), satisfying the Fokker–Planck equation,

2.4 $$\frac{\partial \psi }{\partial t^{*}}+\nabla \cdot (u\psi )+\nabla_{p}\cdot (\Omega^{*}\psi )=D_{r}^{*}\nabla_{p}^{2}\psi ,$$

where **p** is the unit orientation vector,

2.5 p=(sin⁡θcos⁡ϕ,sin⁡θsin⁡ϕ,cos⁡θ),

for *θ*∈[0, *π*] and *ϕ*∈[0, 2*π*) shown in figure 1*b*, **∇**_**p**_ represents differentiation in orientation space and D*_r_ is the rotational diffusion constant. The particle rotation due to shear is given by

2.6 $$\Omega^{*}=(I-pp)\cdot (\alpha_{0}e^{*}+\omega^{*})\cdot p,$$

where e* = (∇*u* + ∇*u*^T^)/2 is the rate of strain tensor, ω* = (∇*u* − ∇*u*^T^)/2 is the vorticity tensor ((∇*u*)_ij_ = ∂u*_i_/∂x*_j_) and *α*_0_ = (r^2^ − 1)/(r^2^ + 1), for particle aspect ratio r, is a measure of slenderness [17]. The orientation distribution function must satisfy periodicity and the normalization condition,

2.7 ψ(ϕ,θ)=ψ(ϕ+π,θ)=ψ(ϕ,θ+2π)

and

2.8 $$\int_{s}\psi \;dp=1,$$

where s is the surface of the unit sphere.

Following Pedley & Kessler [22] (and the prior work of Batchelor [20] and Hinch & Leal [41]), the enhanced stress tensor for a suspension of inactive particles is

2.9 $$\sigma^{*}=\sigma_{I}^{*}+\sigma_{D}^{*}+\sigma_{P}^{*},$$

where the Newtonian contribution is

2.10 $$\sigma_{I}^{*}=-P^{*}I+2\mu^{*}e^{*},$$

for pressure P*, identity matrix **I** and viscosity μ*. Here, σ*_D_ is the extra stress due to the rotary diffusion of the particles and σ*_P_ represents the interaction of the particles with the fluid, given by

2.11 $$\sigma_{D}^{*}=2\mu^{*}\Phi D_{r}^{*}\alpha_{r}\int_{s}\left(pp-\frac{I}{3}\right)\psi \;dp$$

and

2.12 $$\begin{array}{rl} \sigma_{P}^{*} & =4\;\mu^{*}\;\Phi [\alpha_{2}\;e^{*}:\int_{s}p\;p\;p\;p\;\psi \;dp+\alpha_{3}\left(e^{*}\cdot \int_{s}p\;p\;\psi \;dp+\int_{s}p\;p\;\psi \;dp\cdot e^{*}\right)\\ & \;+\alpha_{4}\;e^{*}\int_{s}\psi \;dp+\alpha_{5}\;I\;e^{*}:\int_{s}p\;p\;\psi \;dp], \end{array}$$

and where *Φ* is the volume fraction of particles. The constants *α*_i_ (i = 2…5,  r) relate to the aspect ratio of the particle and can be represented in terms of ellipsoidal integrals [17,20], which are detailed in equations (S1)–(S5) in electronic supplementary material, S1. For reference, Holloway *et al.* [43] have summarized this system of equations for particle suspensions in concise notation. All results in this paper include the full particle stresses ***σ***_D_ and ***σ***_P_.

Because the width and depth of the channel are comparable and much smaller than the channel length, we use a lubrication approximation, introducing the dimensionless parameter *δ* = h*/L*≪1 to allow detailed analysis of the significant hydrodynamic interactions. The variables are scaled via

2.13 $$\begin{array}{rl} & u^{*}=\frac{G^{*}{h^{*}}^{2}}{\mu^{*}}u,\;v^{*}=\frac{\delta G^{*}{h^{*}}^{2}}{\mu^{*}}v,\;w^{*}=\frac{\delta G^{*}{h^{*}}^{2}}{\mu^{*}}w\\ \;\mathrm{and}\;\; & x^{*}=\frac{h^{*}}{\delta }x,\;y^{*}=h^{*}y,\;z^{*}=h^{*}z,\;P^{*}=\frac{G^{*}h^{*}}{\delta }P, \end{array}\}$$

where G* is a reference pressure gradient. For steady flow, G* is the pressure gradient in the channel and for oscillating flow it is equal to the amplitude of the oscillating pressure gradient. The full dimensionless models for steady and oscillatory flow are described in the relevant sections.

Finally, the degree of orientation in a system can be described by an orientation parameter, S, calculated as

2.14 $$\begin{array}{rl} S & =\langle p_{x^{*}}^{2}\rangle -\langle p_{y^{*}}^{2}\rangle ,\\ & =\langle \sin^{2}\theta \cos^{2}\phi \rangle -\langle \sin^{2}\theta \sin^{2}\phi \rangle , \end{array}$$

for a 3D suspension where

2.15 $$\langle ∙\rangle =\int_{0}^{2\pi }\int_{0}^{\pi }∙\;\psi \sin\theta \;d\theta d\phi .$$

Note that this is different from the definition used by McLachlan *et al.* [10],

2.16 $$S=\frac{1}{2}\left(3\int_{s}\cos^{2}\theta_{S}\;\psi \;dp-1\right),$$

where

$$\theta_{S}=\arccos(|\sin\theta \cos\phi |).$$

The expression (2.16) relates only to uniaxial samples such as planar membranes in which *ψ* = *ψ*(*θ*_S_, t*) [9,44], rather than flow spectroscopy in which the orientation distribution is biaxial, i.e. *ψ* = *ψ*(*θ*, *ϕ*, t*). McLachlan *et al.*'s [10] use of this formula is therefore inaccurate in this context.

Equations (2.1)–(2.12) constitute the full dimensional model for variables u*(x*, t*) and *ψ*(*ϕ*, *θ*, t*) which will be solved numerically. The steady flow system is described in §3 and the oscillatory system in §4.

## 3. Steady flow

The first problem we consider is flow forced by a constant pressure gradient G*. §3a contains the Fokker–Planck equation and §3b contains the model for the flow. The full coupled problem is solved by iterating between the numerical methods described in §3a,b.

### (a) Model for the orientation distribution function

The particles in the flow are small enough that each particle at a point in space is subject to an approximately linear velocity field in the local frame, and hence a constant shear rate ${\dot{\gamma }}^{*}=\sqrt{{\left(\nabla^{*}u^{*}\right)}^{2}+{\left(\nabla^{*}{u^{*}}^{T}\right)}^{2}}$. Therefore, the orientation distribution of the bacteriophage is formulated at each point in space independently; fluid flow influences orientation distribution only via the local Péclet number. We describe the model and its numerical discretization below. Applying lubrication theory, assuming *α*_0_ = 1 and investigating steady-state behaviour, the Fokker–Planck equation reduces to

3.1 $$\frac{1}{6}\Lambda (\psi )=P_{e}\Upsilon (\psi ),$$

where $P_{e}={\dot{\gamma }}^{*}/6D_{r}^{*}$ is the local Péclet number and

3.2 $$\Lambda (\psi )=\frac{1}{\sin\theta^{\prime }}\frac{\partial }{\partial \theta^{\prime }}\left(\sin\theta^{\prime }\frac{\partial \psi }{\partial \theta^{\prime }}\right)+\frac{1}{\sin^{2}\theta^{\prime }}\frac{\partial^{2}\psi }{\partial \phi^{\prime 2}}$$

and

3.3 $$\Upsilon (\psi )=\frac{\sin\phi^{\prime }\cos\phi^{\prime }}{\sin\theta^{\prime }}\frac{\partial }{\partial \theta^{\prime }}\left(\sin^{2}\theta^{\prime }\cos\theta^{\prime }\;\psi \right)-\frac{\partial }{\partial \phi^{\prime }}\left(\sin^{2}\phi^{\prime }\;\psi \right),$$

where *θ*′ and *ϕ*′ describe the local coordinates at each point in space, which are calculated by rotating *ϕ* and *θ* away from the laboratory frame by an angle $\beta =\arccos(|\partial u^{*}/\partial y^{*}|/{\dot{\gamma }}^{*})$. The above equation is similar to the spatially uniform case presented by Strand *et al*. [12] (see also Bird *et al.* [45]), with the complication of requiring a local coordinate system.

The Fokker–Planck equation (3.1) is spatially discretized via spherical harmonics [10,12,45]. Assume the solution takes the form

3.4 $$\psi (\phi^{\prime },\theta^{\prime })=\sum_{n=0}^{N}\sum_{m=0}^{n}\left(A_{0\;n}^{m}P_{n}^{m}(\cos\theta^{\prime })\cos(m\phi^{\prime })+A_{1\;n}^{m}P_{n}^{m}(\cos\theta^{\prime })\sin(m\phi^{\prime })\right),$$

where P^m^_n_ are the associated Legendre polynomials. By substituting (3.4) into equation (3.1), *Λ* and *Υ* are given by

3.5 $$\begin{array}{rl} & \Lambda (P_{n}^{m}(\cos\theta^{\prime })\cos(m\phi^{\prime }))=-n(n+1)P_{n}^{m}(\cos\theta^{\prime })\cos(m\phi^{\prime }), \end{array}$$

3.6 $$\begin{array}{rl} & \Lambda (P_{n}^{m}(\cos\theta^{\prime })\sin(m\phi^{\prime }))=-n(n+1)P_{n}^{m}(\cos\theta^{\prime })\sin(m\phi^{\prime }), \end{array}$$

3.7 $$\begin{array}{rl} & \Upsilon (P_{n}^{m}(\cos\theta^{\prime })\cos(m\phi^{\prime }))=-\sum_{p=m-2}^{m+2}\sum_{q=n-2}^{n+2}a_{n,q}^{m,p}P_{n}^{m}(\cos\theta^{\prime })\sin(m\phi^{\prime })\;m\geq 0 \end{array}$$

3.8 $$\begin{array}{rl} \;\mathrm{and}\; & \Upsilon (P_{n}^{m}(\cos\theta^{\prime })\sin(m\phi^{\prime }))=\sum_{p=m-2}^{m+2}\sum_{q=n-2}^{n+2}a_{n,q}^{m,p}P_{n}^{m}(\cos\theta^{\prime })\cos(m\phi^{\prime }),\;m>0. \end{array}$$

The first two results follow from the definition of a spherical harmonic, i.e. the solution to the spherical part of Laplace's equation on a sphere; the other two are a linear combination of spherical harmonics where the seven non-zero constants a^m,p^_n,q_, reproduced from Bird [45] and Strand *et al*. [12], are given in electronic supplementary material S2. The resulting system of equations for A^m^_0 n_ and A^m^_1 n_, found by equating coefficients of sine and cosine, is

3.9 $$\frac{q(q+1)}{6}A_{0\;q}^{p}=-P_{e}\sum_{n=0}^{N}\sum_{m=0}^{n}a_{n,q}^{m,p}A_{1\;n}^{m}$$

and

3.10 $$\frac{q(q+1)}{6}A_{1\;q}^{p}=P_{e}\sum_{n=0}^{N}\sum_{m=0}^{n}a_{n,q}^{m,p}A_{0\;n}^{m},$$

for integer N, where q = 0, 2, …, N and p = 0, 2, …, q. Here, A^0^_0 0_ = 1/4*π* to satisfy the normalization condition, and we have that A^0^_1 n_ = 0 for all n. Due to particle symmetry, if n or m are odd then A^m^_0 n_ = A^m^_1 n_ = 0.

The system (3.9)–(3.10) is solved numerically by setting up a matrix of equations for A^p^_0 q_ and A^p^_1 q_ and using a direct solver (backslash) in matlab.

### (b) Model for the velocity

Under the lubrication theory approximation, the flow problem can be expressed entirely as an elliptic partial differential equation for u(y, z) where the coefficients depend on moments of the orientation distribution *ψ*(*ϕ*, *θ*;y, z).

Scaling the system, via (2.13), neglecting terms O(δ) and smaller, the continuity equation (2.2) is unchanged and the Navier–Stokes equations (2.1) reduce to

3.11 $$\begin{array}{rl} -1 & =\frac{\partial }{\partial y}(\left\{1+4\Phi \left[\alpha_{2}\int_{s}p_{1}^{2}p_{2}^{2}\psi \;dp+\frac{\alpha_{3}}{2}\int_{s}(p_{1}^{2}+p_{2}^{2})\psi \;dp+\frac{\alpha_{4}}{2}\right]\right\}\frac{\partial u}{\partial y}\\ & \;+\;4\Phi \left[\alpha_{2}\int_{s}p_{1}^{2}p_{2}p_{3}\psi \;dp+\frac{\alpha_{3}}{2}\int_{s}p_{2}p_{3}\psi \;dp\right]\frac{\partial u}{\partial z})\\ & \;+\frac{\partial }{\partial z}(\left\{1+4\Phi \left[\alpha_{2}\int_{s}p_{1}^{2}p_{3}^{2}\psi \;dp+\frac{\alpha_{3}}{2}\int_{s}(p_{1}^{2}+p_{3}^{2})\psi \;dp+\frac{\alpha_{4}}{2}\right]\right\}\frac{\partial u}{\partial z}\\ & \;+\;4\Phi \left[\alpha_{2}\int_{s}p_{1}^{2}p_{2}p_{3}\psi \;dp+\frac{\alpha_{3}}{2}\int_{s}p_{2}p_{3}\psi \;dp\right]\frac{\partial u}{\partial y})\\ & \;+\frac{2\Phi \alpha_{r}}{P_{G}}\left(\frac{\partial }{\partial y}\int_{s}p_{1}p_{2}\psi \;dp+\frac{\partial }{\partial z}\int_{s}p_{1}p_{3}\psi \;dp\right), \end{array}$$

for global Péclet number P_G_ = G*h*/D*_r_μ*. The y- and z-components of the dimensionless Navier–Stokes equations enforce P = P(x) and so the pressure gradient reduces to −1. The boundary conditions (2.3) become

3.12 $$u(\pm \bar{W},z)=0,\;u(y,\pm 1)=0,$$

where $\bar{W}=W^{*}/h^{*}$. Equation (3.11), along with boundary conditions (3.12), are solved numerically using a finite difference scheme and constructing a matrix of equations from the resulting problem. The numerical solution to the Newtonian and suspension models are described in electronic supplementary material, S3, and the iterative process is described in electronic supplementary material, S4.

To optimize the efficiency of the above processes, the convergence of the spherical harmonic solution and the finite difference approximation are investigated for a range of spherical harmonic modes N and finite-difference grid spacings Y and Z. The full details and related figures are presented in electronic supplementary material, S3; we find that N = 10, Y = 150 and Z = 120 are sufficient for convergence. We will discuss the results in §5.

## 4. Oscillatory flow

The next flow system of interest consists of an oscillating pressure gradient where the length is still much larger than its width and depth but a smaller volume of fluid oscillates back and forth. The pressure gradient is now defined as

4.1 $$\frac{\partial P^{*}}{\partial x^{*}}=G^{*}\exp(i\;\omega^{*}t^{*}),$$

for frequency of oscillations ω*. The dimensional scalings are given by equation (2.13), and the time is scaled against the frequency of oscillations as t* = t/ω*.

The model for the time-dependent Fokker–Planck equation is given in §4a and the model for the velocity profile is given in §4b. The full coupled problem comes from iterating numerically between 4a,b over each time step.

### (a) Model for the orientation distribution

Assuming the particles are small enough that at each point in the channel they are subjected to a spatially linear velocity field and thus a shear rate dependent only on time ${\dot{\gamma }}^{*}={\dot{\gamma }}^{*}(t^{*})$; the orientation distribution is again formulated at each point in space independently. Following §3a, the Fokker–Planck equation is

4.2 $$\frac{\partial \psi }{\partial \tau }=\frac{1}{6}\Lambda (\psi )-P_{e}(\tau )\Upsilon (\psi ),$$

where $P_{e}(t^{*})={\dot{\gamma }}^{*}/6D_{r}^{*}$ is the local Péclet number, t* = *τ*/6D*_r_ and *Λ* and *Υ* are given by (3.2) and (3.3), respectively; note the introduction of a local time-scale *τ*.

The orientation distribution is discretized spatially in the basis of spherical harmonics,

4.3 $$\psi (\phi^{\prime },\theta^{\prime },\tau )=\sum_{n=0}^{N}\sum_{m=0}^{n}\left(A_{0\;n}^{m}(\tau )P_{n}^{m}(\cos\theta^{\prime })\cos(m\phi^{\prime })+A_{1\;n}^{m}(\tau )P_{n}^{m}(\cos\theta^{\prime })\sin(m\phi^{\prime })\right).$$

This is then substituted into (4.2), where *Λ* and *Υ* are given by (3.5)–(3.8). Equating coefficients of sine and cosine, the resulting system of ordinary differential equations for A^m^_0 n_ and A^m^_1 n_ is

4.4 $$\frac{dA_{0\;q}^{p}}{d\tau }=-\frac{q(q+1)}{6}A_{0\;q}^{p}-P_{e}(\tau )\sum_{n=0}^{N}\sum_{m=0}^{n}a_{n,q}^{m,p}A_{1\;n}^{m}$$

and

4.5 $$\frac{dA_{1\;q}^{p}}{d\tau }=-\frac{q(q+1)}{6}A_{1\;q}^{p}+P_{e}(\tau )\sum_{n=0}^{N}\sum_{m=0}^{n}a_{n,q}^{m,p}A_{0\;n}^{m},$$

for integer N, where q = 0, 2, …, N and p = 0, 2, …, q. Here, A^0^_0 0_(*τ*) = 1/4*π* to satisfy the normalization condition, A^0^_1 n_ = 0 for all n and A^m^_0 n_ = A^m^_1 n_ = 0 for odd n, m.

The ordinary differential equations (4.4) and (4.5) are solved numerically using a second-order explicit Improved Euler method by first writing the equations in matrix form as described in §3a.

### (b) Model for the velocity

Again using a lubrication theory approximation, the velocity problem can be expressed as a time-dependent partial differential equation where coefficients of u(y, z, t) depend upon moments of the orientation distribution function *ψ*(*ϕ*, *θ*;y, z, t).

Scaling the Navier–Stokes and continuity equations, (2.1) and (2.2) respectively, retaining terms larger than O(δ), the continuity equation is unchanged and the dimensionless Navier–Stokes equations become

4.6 $$\begin{array}{rl} \alpha^{2}\frac{\partial u}{\partial t} & =-\exp(it)+\frac{2\Phi \alpha_{r}}{P_{G}}\left(\frac{\partial }{\partial y}\int_{s}p_{1}p_{2}\psi \;dp+\frac{\partial }{\partial z}\int_{s}p_{1}p_{3}\psi \;dp\right)\\ & \;+\frac{\partial }{\partial y}(\left\{1+4\Phi \left[\alpha_{2}\int_{s}p_{1}^{2}p_{2}^{2}\psi \;dp+\frac{\alpha_{3}}{2}\int_{s}(p_{1}^{2}+p_{2}^{2})\psi \;dp+\frac{\alpha_{4}}{2}\right]\right\}\frac{\partial u}{y}\\ & \;+\;4\Phi \left[\alpha_{2}\int_{s}p_{1}^{2}p_{2}p_{3}\psi \;dp+\frac{\alpha_{3}}{2}\int_{s}p_{2}p_{3}\psi \;dp\right]\frac{\partial u}{\partial z})\\ & \;+\frac{\partial }{\partial z}(\left\{1+4\Phi \left[\alpha_{2}\int_{s}p_{1}^{2}p_{3}^{2}\psi \;dp+\frac{\alpha_{3}}{2}\int_{s}(p_{1}^{2}+p_{3}^{2})\psi \;dp+\frac{\alpha_{4}}{2}\right]\right\}\frac{\partial u}{\partial z}\\ & \;+\;4\Phi \left[\alpha_{2}\int_{s}p_{1}^{2}p_{2}p_{3}\psi \;dp+\frac{\alpha_{3}}{2}\int_{s}p_{2}p_{3}\psi \;dp\right]\frac{\partial u}{\partial y}), \end{array}$$

for global Péclet number P_G_ and Womersley number *α*^2^ = ω*h*^2^ρ*/μ*. It is assumed the system is initially at rest, accounted for by altering the dimensionless pressure gradient to ∂P/∂x=exp⁡(i(t+π/2)).

Equation (4.6) is solved via an alternating direction implicit (ADI) method in which the time step is split in half and one spatial variable is discretized in each time step [46]; mixed derivatives are treated explicitly throughout this analysis. As in §3, a matrix equation is constructed for each time step and solved numerically; the details of this method can be found in electronic supplementary material, S5, and a description of the iterative procedure is described in electronic supplementary material, S6.

A numerical convergence study was undertaken, detailed in electronic supplementary material, S5. As in §3, Y = 150 and Z = 120 are satisfactory in the finite difference scheme and T = 1200 time steps ensure convergence.

## 5. Results

The velocity, orientation parameter and linear dichroism signal are compared for a range of particle number density, channel dimensions and pressure gradient. We will refer to the standard parameter set for these quantities as: channel half-depth h* = 3 × 10^−4^ m, half-width W* = 1 × 10^−3^ m, fixed pressure gradient G* ≈ 4.4 × 10^3^ Pa m^−1^, particle number density n*_d_ = 7.33 × 10^17^ phage m^−3^ and frequency of oscillations ω* = 10 rad s^−1^ (table 1(a)). Using a particle length of a* = 800 nm, the value n*_d_a*^3^ = 0.3753 < 1 and so we argue that the approximation of a dilute regime is reasonable. We will also examine the effect of higher particle concentrations, which is beyond the strict regime of validity of dilute theory but with the aim of providing insight into this tractable regime. The dimensionless groups are detailed in table 1(c) and are subject to change depending on their composition of dimensional values; we do not directly vary the dimensionless groups. All other parameters are unchanged (table 1(b)).

**Table 1.** Summary of the standard parameter set split into three categories: (*a*) the parameters that vary throughout the results, (*b*) the fixed parameters and physical constants and (*c*) the dimensionless groups.

(*a*) variable parameters				

parameter	description	standard value	range	units
h*	channel half-depth	3 × 10^−4^	2 × 10^−4^–4 × 10^−3^	m
W*	channel half-width	1 × 10^−3^	0.4 × 10^−3^–2.5 × 10^−3^	m
n*_d_	number density	7.33 × 10^17^	1 × 10^16^–1 × 10^20^	phage m^−3^
G*	pressure gradient	4.4 × 10^3^	0.4 × 10^3^–10 × 10^3^	Pa m^−1^
ω*	frequency of oscillations	10	5–70	rad s^−1^
(*b*) fixed parameters and physical constants				

parameter	description	value	units	
a*	particle length	8 × 10^−7^	m	
b*	particle width	6 × 10^−9^	m	
V*_c_	particle volume	1.21 × 10^−21^	m^3^	
T*	temperature	295	K	
μ*	viscosity (water)	9.5 × 10^−4^	Pa s	
ρ*	fluid density (water)	1 × 10^3^	kg m^−3^	
D*_r_	rotational diffusion coefficient	40.65	s^−1^	
m*_w_	particle molecular weight	2 × 10^7^	g mol^−1^	
ϵ*	extinction coefficient	0.38	m^2^ g^−1^	
K*_B_	Boltzmann constant	1.38 × 10^−23^	kg m^2^ K^−1^ s^−2^	
N*_A_	Avogadro constant	6 × 10^23^	phage mol^−1^	
(*c*) dimensionless groups				

parameter	description	definition	value	
*Φ*	volume fraction	V*_c_n*_d_	1.11 × 10^−5^	
P_G_	Péclet number (global)	G*h*/D*_r_μ*	34.17	
*α*^2^	Womersley number	ω*h*^2^ρ*/μ*	18.95	

The notation $\bar{∙}$ and ${\bar{∙}}^{y^{*}}$ represent the spatial average and the width average for steady flow, respectively,

5.1 $$\bar{\;∙\;}=\frac{1}{4W^{*}h^{*}}\int_{-W^{*}}^{W^{*}}\int_{-h^{*}}^{h^{*}}∙dy^{*}\;dz^{*}\;\mathrm{and}\;{\bar{\;∙\;}}^{y^{*}}=\frac{1}{4W^{*}}\int_{-W^{*}}^{W^{*}}∙\;dy^{*}.$$

The linear dichroism signal is assumed proportional to the particle number density and is calculated as the root mean square (denoted as S_rms_) of the orientation parameter across the depth of the channel. The signal is given by

5.2 $$LD=\frac{\epsilon^{*}m_{w}^{*}n_{d}^{*}}{N_{A}^{*}}S_{\text{rms}},$$

where N*_A_ is the Avogadro constant ( ≈ 6 × 10^23^ phage mol^−1^), m*_w_ is the molecular weight of M13 bacteriophage (m*_w_ ≈ 2 × 10^7^ g mol^−1^ from Linear Diagnostics Ltd) and ϵ* is the extinction coefficient (m^2^ g^−1^), which is a measure of the absorption of light through the sample [7]. The extinction coefficient is taken as 0.38 m^2^ g^−1^ at light wavelength 269 nm [47–49]. A number of the parameters in table 1 and their calculation are discussed in electronic supplementary material, S7. As a direct comparison with steady flow, the maximum value of the orientation parameter in the oscillating channel is presented.

The computational walltime for this problem was large, especially for oscillating flow. The numerical solution was parallelized using *parfor* in matlab by splitting the solution to the Fokker–Planck equation, solved at each point in space, across five cores on a workstation (2017 Lenovo Thinkstation P710; Intel(R) Xeon(TM) E5-2646 CPU @ 2.40 GHz; 128 GB 2400 MHz RDIMM RAM). The runtime varied between 14 and 20 h for a single parameter tuple (ordered set), consisting of the parameters detailed in table 1(a); 175 tuples were calculated for the oscillating results.

### (a) Steady pressure gradient

**Figure 2.**
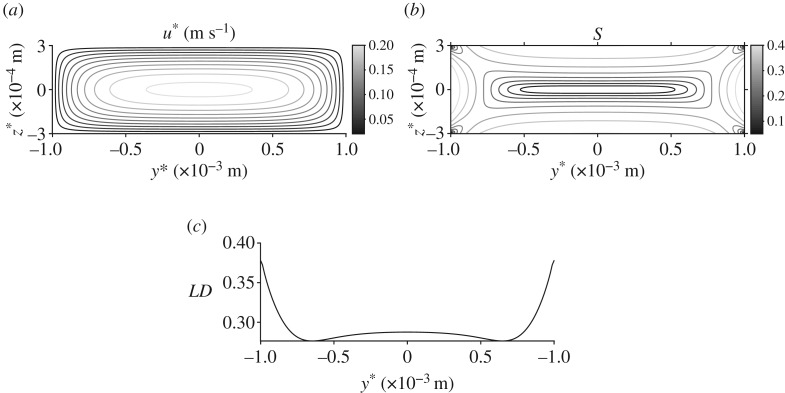
Velocity field, orientation parameter and predicted linear dichroism signal for steady flow and the standard parameter set. (*a*) Velocity profile, (*b*) orientation parameter and (*c*) linear dichroism signal.

Figure 2 depicts the velocity profile, orientation parameter and linear dichroism signal for steady flow through the cross section of the channel, with the standard parameter set. As may be expected from the Newtonian solution [50], the velocity u* increases from zero at the channel walls to a maximum at the centre point (figure 2*a*), corresponding to a minimum in the orientation parameter S (figure 2*b*). Associated with the large shear rate, the linear dichroism signal is largest near the channel walls and decreases to a local minimum at the centre (figure 2*c*); there is an increase at the middle width of the channel (y* = 0) where larger shear near the walls at z* = ± h* increases the orientation parameter. Local minima also exist at the channel corners.

**Figure 3.**
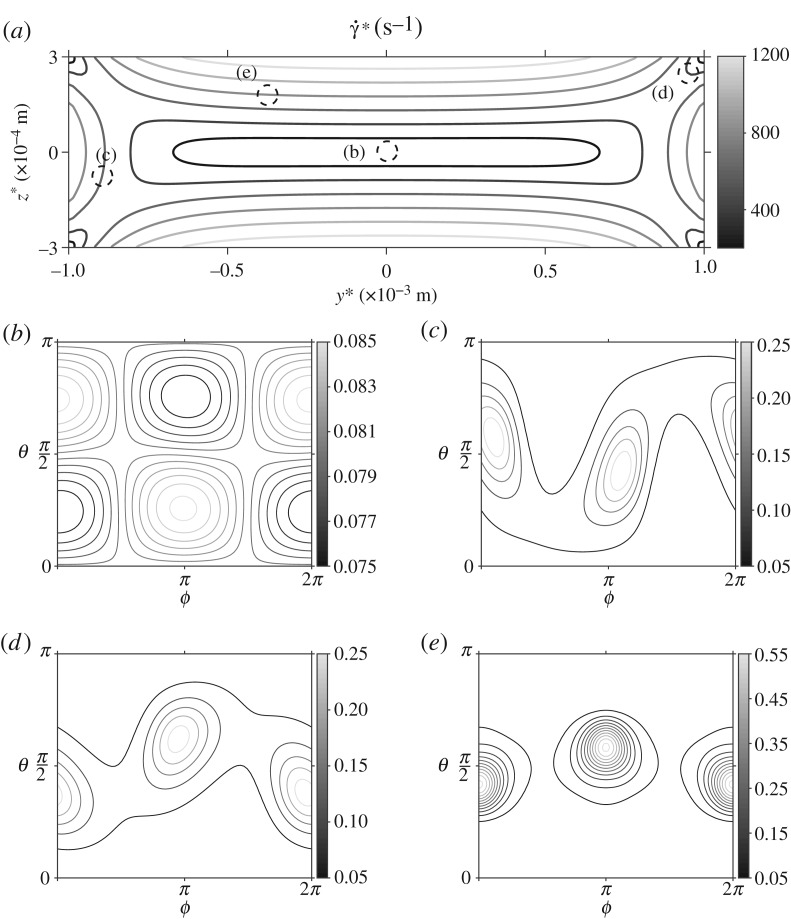
The orientation distribution function *ψ*, in orientation space *ψ*∈ [0,  2*π*], *θ*∈[0,  *π*), at different points in the cross-sectional face of the flow using the standard parameter set. (*a*) The shear rate ${\dot{\gamma }}^{*}$ in the cross-sectional face of the channel. The dashed circles, with corresponding labels, show the points in the flow where *ψ* has been evaluated: (*b*) y* = 0 m, z* = 0 m, (*c*) y* = − 0.4 × 10^−3^ m, z* = 2 × 10^−4^ m, (*d*) y* = 0.95 × 10^−3^ m, z* = 2.75 × 10^−4^ m and (*e*) y* = − 0.8 × 10^−3^ m, z* = − 1 × 10^−4^ m.

The orientation distribution function *ψ* is displayed for varying points in the channel cross section (figure 3). At the centre of the channel where the shear rate is zero (figure 3*b*) there are no biasing effects on the particles and so *ψ* is almost uniform in orientation space. Close to the walls at y* = ± W* the behaviour of *ψ* is similar (figure 3*c*,*d*); the particles preferentially align in the flow direction, for orientation angles *ϕ* = 0,  *π* and 2*π* and *θ* = *π*/2. Close to the corner of the channel this behaviour is more prominent. Near z* = h* (figure 3*e*), the M13 bacteriophage are more likely to be aligned at *ϕ* = 0 and *π* (note that the particles have no polarity so the two angles are equivalent) and *θ* = *π*/2.

**Figure 4.**
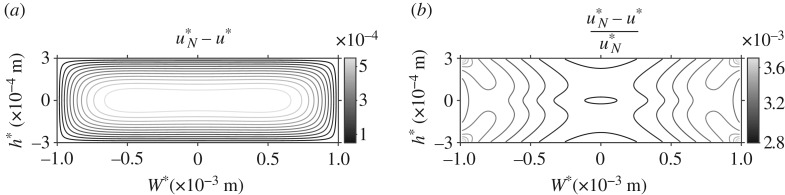
The difference between the Newtonian and suspension flow fields in the cross-sectional face of the channel. (*a*) Depicts the actual difference u*_N_ − u* and (*b*) depicts the difference relative to the magnitude of the Newtonian velocity (u*_N_ − u*)/u*_N_.

The flow profile accounting for a particle suspension at standard concentration is compared with the Newtonian profile u*_N_ for the same flow set-up in figure 4. The actual difference shows that the profile for both flow systems is qualitatively the same; the suspension model produces a lower velocity however the change is small (figure 4*a*). To confirm that this change is small, the reduction in velocity relative to the magnitude of the Newtonian velocity is also calculated in figure 4*b* and the change is on the order of 10^−3^. This effect has been investigated for higher particle concentrations (electronic supplementary material, figure S4); above n*_d_ ≈ 10^18^ phage m^−3^ there is a greater reduction in the flow.

**Figure 5.**
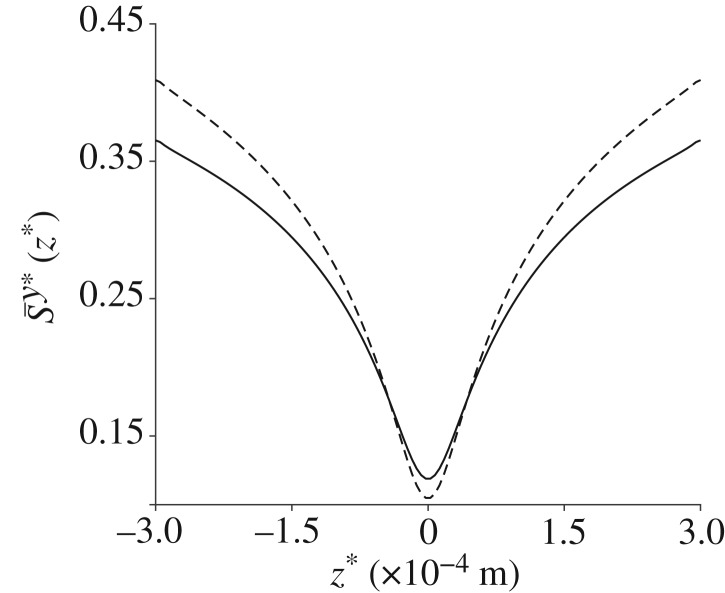
A comparison between the width-averaged orientation parameter calculated in the current work (solid line) and the definition used by McLachlan *et al.* [10] (dashed line), for the standard parameter set.

We compare the width-averaged orientation parameter calculated in this work (solid line) with equation (2.16) (dashed line) in figure 5; while both definitions show maxima and minima of the orientation parameter at the same point in the channel, McLachlan *et al.*'s definition for the orientation parameter [10] over-predicts the orientation in general.

**Figure 6.**
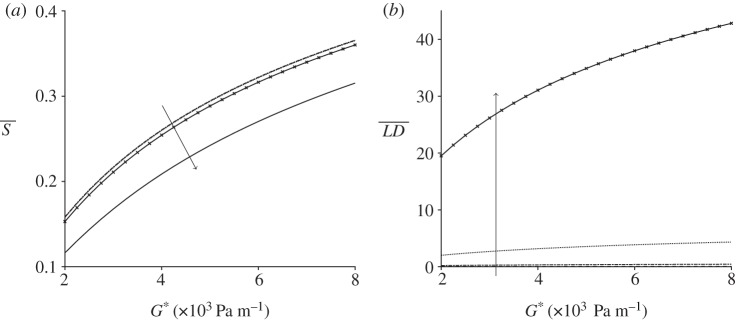
The orientation parameter and linear dichroism signal for steady flow with increasing pressure gradient number density. (*a*) The orientation parameter S. (*b*) The linear dichrosim signal LD. Results for several particle number densities are shown: n*_d_ = 1 × 10^16^ phage m^−3^ (dashed line), n*_d_ = 1 × 10^17^ phage m^−3^ (dashed-dotted line), and n*_d_ = 1 × 10^18^ phage m^−3^ (dotted line) and n*_d_ = 1 × 10^19^ phage m^−3^ (crossed line), in (*a*) the additional solid line is n*_d_ = 1 × 10^20^ phage m^−3^; the arrows denote s increasing number density. The other parameters are h* = 3 × 10^−4^ m and W* = 1 × 10^−3^ m.

In figure 6, the impact of the pressure gradient G* and particle number density n*_d_ on the spatially averaged orientation parameter S and linear dichroism signal LD is examined. Increasing the number density reduces the orientation parameter; the particle effects become significant above n*_d_ ≈ 1 × 10^18^ phage m^−3^. This effect is shown in more detail in electronic supplementary material, figure S4, in which the spatially averaged velocity profile and orientation parameter are calculated for varying number density. The velocity and orientation parameter are reduced approximately 30 and 19%, respectively, over three orders of magnitude. Increasing the number density significantly increases the linear dichroism signal due to their linear relationship (figure 6*b*).

**Figure 7.**
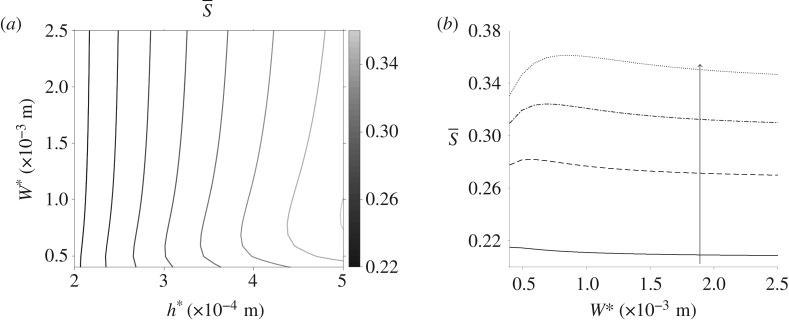
The orientation parameter changing with channel dimensions for steady flow. (*a*) S for a range of channel width and depth, (*b*) S changing with increasing channel width W* for multiple small channel width values: h* = 2 × 10^−4^ m (solid line), h* = 3 × 10^−4^ m (dashed line), h* = 4 × 10^−4^ m (dashed-dotted line) and h* = 5 × 10^−4^ m (dotted line) and the arrow denotes increasing channel width. Here, G* = 4.4 × 10^3^ Pa m^−1^ and n*_d_ = 7.33 × 10^17^ phage m^−3^.

**Figure 8.**
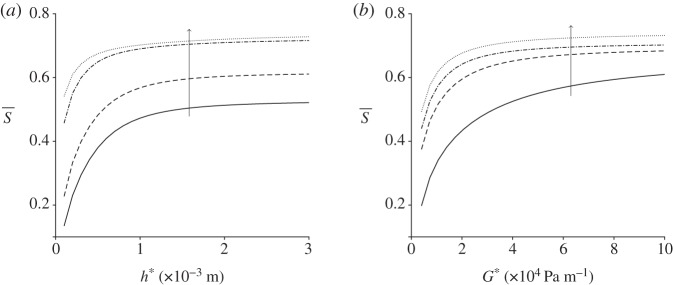
Limit of S with increasing channel depth and pressure gradient for steady flow. (*a*) S with varying channel depth h* for a range of pressure gradients: G* = 5 × 10^3^ Pa m^−1^ (solid line), G* = 1 × 10^4^ Pa m^−1^ (dashed line), G* = 5 × 10^4^ Pa m^−1^ (dot-dashed line) and G* = 10 × 10^4^ Pa m^−1^ (dotted line). The arrow denotes increasing G*. (b) S varying with pressure gradient G* for a range of channel depths: h* = 2 × 10^−4^ m (solid line), h* = 6 × 10^−4^ m (dashed line), h* = 1 × 10^−3^ m (dashed-dotted line) and h* = 4 × 10^−3^ m (dotted line). The arrow denotes increasing h*.

The spatially averaged orientation parameter is calculated for a range of channel widths and depths in figure 7; the other parameters, including pressure gradient, remain fixed. The channel depth has a large impact on S; the orientation parameter increases as the depth is increased. The channel width has limited effect, unless the two dimensions are comparable (figure 7*b*). For channel widths below W* = 1 × 10^−3^ m increasing the channel width increases the orientation parameter; this effect reverses as W* grows. Finally, the upper asymptote of the spatially averaged orientation parameter is investigated for large pressure gradient and channel depth (figure 8). In both cases, S quickly tends towards a constant value, $\bar{S}\approx 0.74$.

### (b) Oscillating pressure gradient

**Figure 9.**
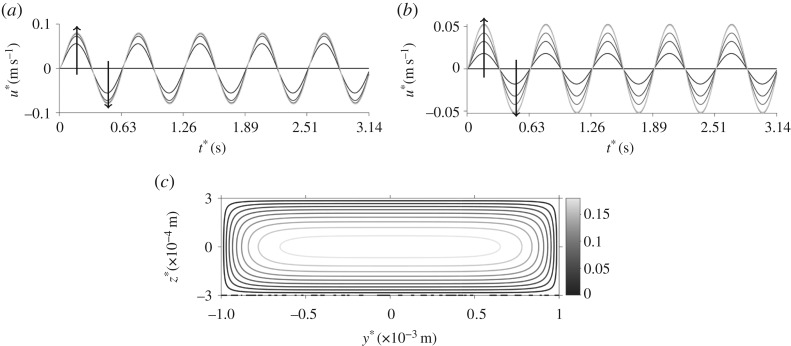
Velocity field for oscillating flow and the standard parameter set. (*a*) The velocity changing over time for multiple y* where z* = 3 × 10^−4^ m. The arrows denote increasing positive values of y*. (*b*) The velocity changing over time for multiple points in z*, where y* = 2 × 10^−3^ m. The arrows denote increasing positive values of z*. (*c*) The velocity field, averaged over half a time period, through the cross-sectional face of the channel.

**Figure 10.**
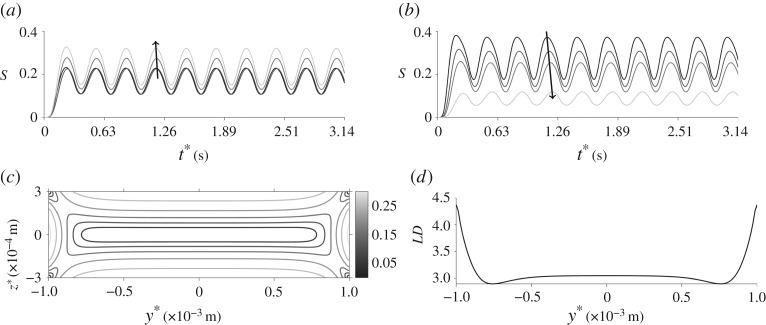
The orientation parameter for oscillating flow and the standard parameter set. (*a*) S evolving in time for different values of z* where y* = 1.9 × 10^−3^ m. The arrow denotes increasing z*. (*b*) S evolving in time for different values of z* where y* = 3.5 × 10^−4^ m. The arrow denotes increasing z*. (*c*) S through the cross-sectional face of the channel, averaged over half a time period. (*d*) LD averaged over half a time period.

The velocity, orientation parameter and linear dichroism signal for oscillatory flow with the standard parameter set are shown in figures 9 and 10.

Figure 9*a*,*b* shows how the velocity changes in time across the width and depth of the channel, respectively (away from edge effects in the other coordinate); the velocity oscillates and is zero at the walls as expected. A time-averaged velocity profile is plotted in figure 9*c*, this is qualitatively similar to the steady velocity profile in figure 2*a* but the magnitude of the velocity is reduced.

The orientation parameter is shown over time and for multiple values of z* in figures 10*a* for y* = 1.9 × 10^−3^ m, and 10*b* for y* = 3.5 × 10^−4^ m. Towards the middle width (figure 10*a*), the orientation parameter increases with the channel depth and in the centre of the channel (figure 10*b*) the opposite occurs. Figure 10*c* displays the time-averaged orientation parameter which agrees well with that produced in steady flow (figure 2*b*); this is also seen for the linear dichroism signal in figure 10*d*. Note here that the peak values of the orientation parameter are similar for steady and oscillatory flow.

**Figure 11.**
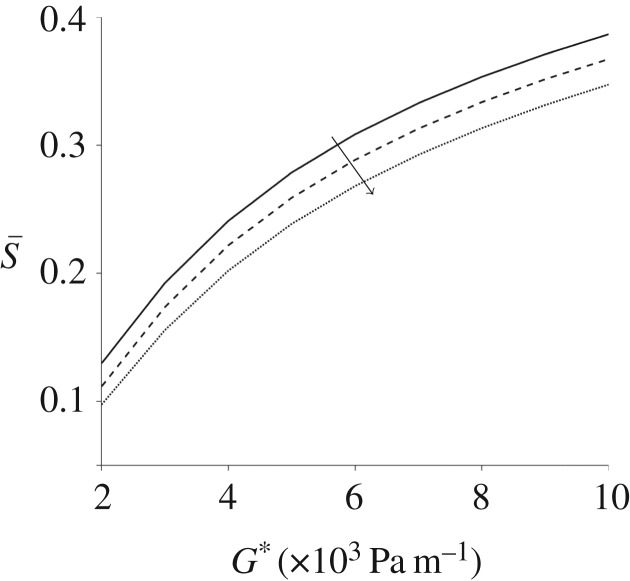
Spatially averaged orientation parameter, for oscillating flow, varying with increasing pressure gradient and for three values of ω*: ω* = 10 rad s^−1^ (solid line), ω* = 20 rad s^−1^ (dashed line) and ω* = 30 rad s^−1^ (dotted line). Here h* = 3 × 10^−4^ m, W* = 1 × 10^−3^ m, n*_d_ = 1 × 10^17^ phage m^−3^ and the arrow denotes increasing frequencies of oscillation.

Next, the spatially averaged orientation parameter S is compared for various pressure gradient and frequencies of oscillation (figure 11). As the pressure gradient G* increases, the orientation parameter increases; there is an indication that the impact of G* on S is reducing as G* becomes large. The impact of increasing the frequency of oscillation ω* is to decrease S, a result seen throughout all figures. The relationship between the pressure gradient and frequencies of oscillation, for a choice of channel depth, is summarized in table 2.

**Table 2.** The spatially averaged orientation parameter for oscillating flow and a range of frequency of oscillations, pressure gradient and channel depth. Throughout these results, n*_d_ = 1 × 10^18^ phage m^−3^ and W* = 2 × 10^−3^ m.

G* (Pa m^−1^)	h* (m)	S (ω* = 10 rad s^−1^)	S (ω* = 100 rad s^−1^)
2 × 10^3^	2 × 10^−4^	0.130	0.042
	4 × 10^−4^	0.193	0.031
4 × 10^3^	2 × 10^−4^	0.241	0.110
	4 × 10^−4^	0.316	0.081
6 × 10^3^	2 × 10^−4^	0.309	0.165
	4 × 10^−4^	0.381	0.122
8 × 10^3^	2 × 10^−4^	0.353	0.207
	4 × 10^−4^	0.424	0.154

**Figure 12.**
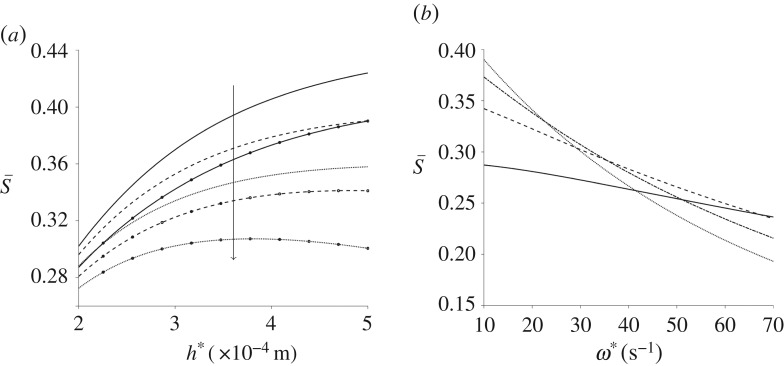
Spatially averaged orientation parameter for oscillating flow, changing with channel dimensions and frequency of oscillation. (*a*) S varying with channel depth for multiple frequencies of oscillation: ω* = 10 rad s^−1^ (solid line), ω* = 20 rad s^−1^ (dashed line) and ω* = 30 rad s^−1^ (dotted line). The lines without markers are for W* = 5 × 10^−4^ m and the lines with markers are for W* = 2 × 10^−3^ m. The arrow denotes increasing frequencies of oscillations. (*b*) S changing with frequency of oscillations for multiple values of h*: h* = 2 × 10^−4^ m (solid line), h* = 3 × 10^−4^ m (dashed line), h* = 4 × 10^−4^ m (dashed-dotted line) and h* = 5 × 10^−4^ m (dotted line) and for W* = 2 × 10^−3^ m. For both figures, G* = 8 × 10^3^ Pa m^−1^ and n*_d_ = 1 × 10^17^ phage m^−3^.

The orientation parameter is calculated for increasing channel depth h* and for a number of frequencies of oscillation and channel width (figure 12). Increasing W*, shown by line markers in figure 12*a*, decreases the orientation parameter in general. The orientation parameter has a non-monotonic response to h*as the frequency of oscillations is increased; for small ω*, increasing h* increases the orientation parameter whereas for large ω* this relationship begins to reverse. This trend is highlighted in figure 12*b*, in which W* = 2 × 10^−3^ m. The relationship between h*, W*, ω* and the orientation parameter S has been summarized in table 3; when ω* is large, a larger reduction in S is seen with increasing channel width.

**Table 3.** The spatially averaged orientation parameter for oscillating flow and a range of channel width, number density and frequencies of oscillations. Throughout these results, G* = 8 × 10^3^ Pa m^−1^ and n*_d_ = 1 × 10^18^ phage m^−3^.

W* (m)	h* (m)	S (ω* = 10 rad s^−1^)	S (ω* = 100 rad s^−1^)
0.5 × 10^−3^	2 × 10^−4^	0.302	0.227
	4 × 10^−4^	0.406	0.217
2 × 10^−3^	2 × 10^−4^	0.287	0.212
	4 × 10^−4^	0.373	0.173

**Figure 13.**
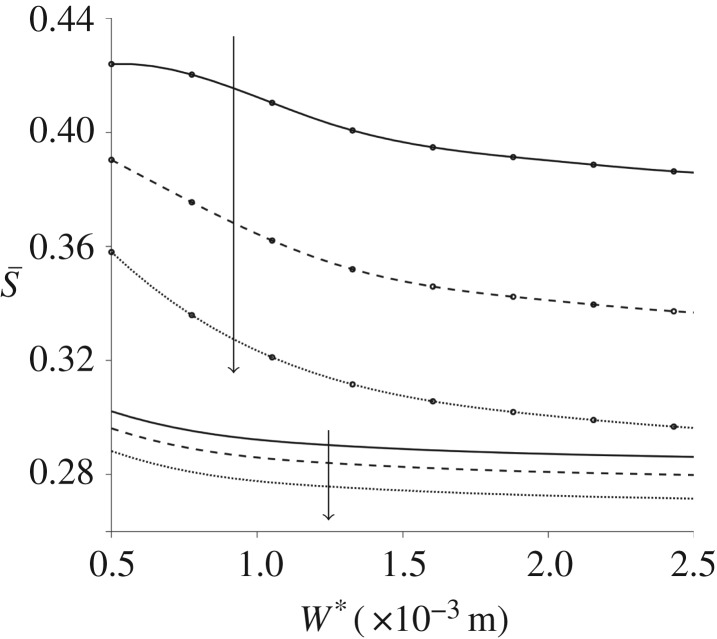
Spatially averaged orientation parameter, for oscillating flow, changing with increasing channel width W*. Three values of the frequency of oscillations are plotted: ω* = 10 rad s^−1^ (solid lines), ω* = 20 rad s^−1^ (dashed lines) and ω* = 30 rad s^−1^ (dotted lines). Two values of h* are also displayed: h* = 2 × 10^−4^ m is shown by plain lines and h* = 5 × 10^−4^ m is shown by the line markers. The arrows denote increasing frequencies of oscillation.

To further investigate these properties, the orientation parameter is plotted for increasing channel width for a range of channel depth and frequencies of oscillations (figure 13). As seen previously, the orientation parameter S increases with channel depth and decreases with increasing channel width. Increasing the frequency of oscillations reduces the orientation parameter in general; for larger channel depths, the decrease in the orientation parameter with ω* is greater.

## 6. Discussion

The coupled pressure-driven flow and orientation dynamics of a dilute suspension of elongated particles has been modelled, to provide a mechanistic underpinning for flow linear dichroism spectroscopy. The model was applied specifically to a prototype hand-held device for waterborne pathogen detection, to explore the effect of changing the channel dimensions and pressure gradient, and to assess the feasibility of oscillatory flow. Mathematically, the problem involved coupling the Navier–Stokes equations, simplified via lubrication theory, to the Fokker–Planck equation for the shear-induced orientation dynamics. Whereas the flow problem involved two spatial independent variables and time, the orientation problem involved two angular independent variables and time, applied at each spatial location in the domain; the latter follows from the assumption that the particles are sufficiently small that they encounter a locally uniform shear rate. The flow and orientation problems were coupled via corrections to the fluid constitutive law involving moments of the orientation distribution, as described by established orientation Brownian suspension mechanics theory.

The system was solved numerically using spherical harmonics for the orientation distribution and a finite difference method for the fluid dynamics, with iterative coupling; the oscillatory problem was temporally discretized with a second-order explicit method for orientation dynamics and an alternating direction implicit method for the fluid dynamics. The oscillatory flow is rather more computationally expensive due to temporal dependence; a parameter set of 175 tuples was explored for the oscillatory flow, with each tuple requiring 14–20 h of walltime on five workstation cores. The dependence of particle orientation on channel width, depth, pressure gradient, particle number density and frequency of oscillation were reported and compared for both the steady and oscillatory systems.

To model linear dichroism, the analysis focused on a single orientation parameter, calculated as the average of the difference between the components of the particle alignment parallel to, and perpendicular to, the flow direction. It was noted that the earlier (homogeneous shear) analysis of McLachlan *et al.* [10] applied an alignment formula based on uniaxial orientation for elongated particles embedded in a membrane. However, because flow linear dichroism produces a biaxial orientation distribution—in other words, there is no rotational symmetry about the flow direction in general. The correct biaxial formula was compared with the uniaxial formula; it was found that the qualitative behaviour was similar, however, the uniaxial definition overestimates the degree of orientation.

In both steady and oscillatory flows, increasing the pressure gradient increases the shear rate and hence the degree of alignment in the system. For steady flow, a plateau was observed close to a – perhaps maximum achievable – value of $\bar{S}=0.75$, above G* = 5 × 10^4^ Pa m^−1^ and h* = 2 × 10^−3^ m, whereas for oscillating flow the mean orientation did not exceed $\bar{S}=0.43$ for the range of parameters analysed. This result does however suggest that oscillatory flow may be a viable method for producing practically useful levels of alignment, which in turn may be valuable in the analysis of small sample volumes. Alignment decays monotonically with driving frequency, however an increase from ω* = 10 rad s^−1^ to 40 rad s^−1^ results in less than a 25% reduction in alignment. Increasing channel height has a significantly greater impact than increasing channel width, although for oscillating flow there is evidence of a local maximum in alignment as channel height is increased. For a given limited sample volume, there is a clear trade-off between channel volume and oscillatory frequency; as channel volume is increased, a higher oscillatory frequency is needed to keep the sample underneath the detection window. These data reported should enable the optimal configuration for a given volume to be calculated.

Signal production was also modelled by multiplying the alignment parameter by the number density of particles; at lower volume fractions the effect of number density on flow and alignment is weak, so the relationship between number density and signal is predicted to be approximately linear. At higher volume fractions (n*_d_  rsim10^18^) the relationship is slightly weaker than linear due to flow and alignment attenuation. While increasing the number density may therefore appear to be a simple way to optimize signal, in practice there are trade-offs with scattering—which was not modelled in the present case—and cost of the reagent. Optimizing the remaining design parameters is therefore important.

Avenues for further work include experimental testing of the predictions of how signal should vary with channel dimensions and pressure gradient, and direct testing of oscillating flow linear dichroism spectroscopy. It will also be important to take account of scattering and hence produce a more accurate model of how number density affects signal production. Other avenues include incorporating flexibility of particles—since the persistence length of the M13 bacteriophage considered here is approximately 1.3 m [51] and the fibres are of length 0.8 m a rigid fibre approximation is reasonable, however, future work could incorporate the flexibility of the fibres. Depending on particle sizes and time scales, migration induced by anisotropic translational diffusivity can be important [42]; including spatial variations in the concentration would, therefore, be an interesting extension to the current problem.

While the present manuscript focused on flows of passive particle suspensions in thin rectangular channels, future work may include investigating active suspensions, which can display collective behaviour [52–55] and superfluidity [56]. The system considered here can be augmented by an extra stress term to account for self-motility [22],

6.1 $$\sigma_{A}^{*}=\alpha_{1}\Phi \int_{s}\left(pp-\frac{I}{3}\right)\psi \;dp,$$

which could model swimming elongated cells such as spermatozoa in pressure-driven flow or complex behaviours such as trapping in shear flow [57].

The dynamics of suspensions of elongated particles provides an enduring subject in fluid mechanics, with novel application areas continuing to be discovered. The challenging task of solving the complex models associated with non-homogeneous shear and time-dependent forcing is now becoming within reach of multicore computing hardware. We hope that the present manuscript provides both useful information in the design and optimization of the technological system considered, and an effective framework which can be applied and extended to other systems in biophysical spectroscopy and cell analysis.

## Supplementary Material

Summary of numerical methods

## Supplementary Material

List of calculated orientation parameter averages for steady flow

## Supplementary Material

List of calculated velocity and orientation parameter averages for oscillating flow

## Data accessibility

Two CSV files, containing the numerical results for each figure for steady and oscillatory flow, are given in the electronic supplementary material. Both CSV files contain dimensionless results and the dimensional results included in this manuscript.

## References

[1] Kim S, Karrila S 2013 Microhydrodynamics: principles and selected applications. Mineola, NY: Dover Publications.

[2] Hill N, Pedley T 2005 Bioconvection. Fluid Dyn. Res. 37, 1–20. (10.1016/j.fluiddyn.2005.03.002)

[3] Creppy A, Plouraboué F, Praud O, Druart X, Cazin S, Yu H, Degond P 2016 Symmetry-breaking phase transitions in highly concentrated semen. J. R. Soc. Interface 13, 20160575 (10.1098/rsif.2016.0575) 27733694 PMC5095218

[4] Guazzelli E, Morris J 2011 A physical introduction to suspension dynamics. Cambridge, UK: Cambridge University Press.

[5] Cheng X, Joseph M, Covington J, Dafforn T, Hicks M, Rodger A 2012 Continuous-channel flow linear dichroism. Anal. Methods 4, 3169–3173. (10.1039/c2ay25513h)

[6] Marrington R, Dafforn T, Halsall D, MacDonald J, Hicks M, Rodger A 2005 Validation of new microvolume Couette flow linear dichroism cells. Analyst 130, 1608–1616. (10.1039/b506149k) 16284659

[7] Daviter T, Chmel N, Rodger A 2013 Circular and linear dichroism spectroscopy for the study of protein–ligand interactions. In *Protein-ligand interactions*, pp. 211–241. Berlin, Germany: Springer. 10.1007/978-1-62703-398-5_8 23729254

[8] Bulheller B, Rodger A, Hirst J 2007 Circular and linear dichroism of proteins. Phys. Chem. Chem. Phys. 9, 2020–2035. (10.1039/b615870f) 17464384

[9] Rodger A, Nordén B 1997 Circular dichroism and linear dichroism. Oxford, UK: Oxford, UK: Oxford University Press.

[10] McLachlan J, Smith D, Chmel N, Rodger A 2013 Calculations of flow-induced orientation distributions for analysis of linear dichroism spectroscopy. Soft Matter 9, 4977–4984. (10.1039/c3sm27419e)

[11] Batchelor G 1971 The stress generated in a non-dilute suspension of elongated particles by pure straining motion. J. Fluid Mech. 46, 813–829. (10.1017/S0022112071000879)

[12] Strand S, Kim S, Karrila S 1987 Computation of rheological properties of suspensions of rigid rods: stress growth after inception of steady shear flow. J. Non-Newton. Fluid 24, 311–329. (10.1016/0377-0257(87)80044-7)

[13] Rasched I, Oberer E 1986 Ff coliphages: structural and functional relationships. Microbiol. Rev. 50, 401. 3540571 10.1128/mr.50.4.401-427.1986 PMC373080

[14] Pacheco-Gómez R, Kraemer J, Stokoe S, England H, Penn C, Stanley E, Rodger A, Ward J, Hicks M, Dafforn T 2011 Detection of pathogenic bacteria using a homogeneous immunoassay based on shear alignment of virus particles and linear dichroism. Anal. Chem. 84, 91–97. (10.1021/ac201544h) 22017566

[15] Kim J *et al.* 2017 Monitoring the orientation of rare–earth–doped nanorods for flow shear tomography. Nat. Nanotechnol. 12, 914 (10.1038/nnano.2017.111) 28650436

[16] Gallagher M, Neal C, Arkill K, Smith D 2017 Model-based image analysis of a tethered Brownian fibre for shear stress sensing. J. R Soc. Interface 14, 20170564 (10.1098/rsif.2017.0564) 29212755 PMC5746567

[17] Jeffery G 1922 The motion of ellipsoidal particles immersed in a viscous fluid. Proc. R. Soc. Lond. A 102, 161–179. (10.1098/rspa.1922.0078)

[18] Ericksen J 1960 Transversely isotropic fluids. Colloid Polym. Sci. 173, 117–122. (10.1007/bf01502416)

[19] Leslie F 1968 Some constitutive equations for liquid crystals. Arc. Ration. Mech. Anal. 28, 265–283. (10.1007/BF00251810)

[20] Batchelor G 1970 The stress system in a suspension of force-free particles. J. Fluid Mech. 41, 545–570. (10.1017/S0022112070000745)

[21] Peterlin A, Stuart H 1939 Zur Theorie der Strömungsdoppelbrechung von Kolloiden und großen Molekülen in Lösung. Z. Phys. 112, 1–19. (10.1007/BF01325633)

[22] Pedley T, Kessler J 1990 A new continuum model for suspensions of gyrotactic micro-organisms. J. Fluid Mech. 212, 155–182. (10.1017/S0022112090001914) 11537107

[23] Kamal M, Mutel A 1989 The prediction of flow and orientation behavior of short fiber reinforced melts in simple flow systems. Polym. Compos. 10, 337–343. (10.1002/pc.750100510)

[24] Leal L, Hinch E 1972 The rheology of a suspension of nearly spherical particles subject to Brownian rotations. J. Fluid Mech. 55, 745–765. (10.1017/S0022112072002125)

[25] Hinch E, Leal L 1973 Time-dependent shear flows of a suspension of particles with weak Brownian rotations. J. Fluid Mech. 57, 753–767. (10.1017/S0022112073001990)

[26] Manhart M 2003 Rheology of suspensions of rigid–rod like particles in turbulent channel flow. J. Non-Newton Fluid. 112, 269–293. (10.1016/S0377-0257(03)00105-8)

[27] Marchioli C, Fantoni M, Soldati A 2010 Orientation, distribution, and deposition of elongated, inertial fibers in turbulent channel flow. Phys. Fluids 22, 033301 (10.1063/1.3328874)

[28] Mortensen P, Andersson H, Gillissen J, Boersma B 2008 Dynamics of prolate ellipsoidal particles in a turbulent channel flow. Phys. Fluids 20, 093302 (10.1063/1.2975209)

[29] Ezhilan B, Saintillan D 2015 Transport of a dilute active suspension in pressure–driven channel flow. J. Fluid Mech. 777, 482–522. (10.1017/jfm.2015.372)

[30] Schiek R, Shaqfeh E 1997 Cross-streamline migration of slender Brownian fibres in plane Poiseuille flow. J. Fluid Mech. 332, 23–39. (10.1017/S0022112096003291)

[31] Park J, Butler J 2009 Inhomogeneous distribution of a rigid fibre undergoing rectilinear flow between parallel walls at high Péclet numbers. J. Fluid Mech. 630, 267–298. (10.1017/S0022112009006545)

[32] Holloway C, Dyson R, Smith D 2015 Linear Taylor–Couette stability of a transversely isotropic fluid. Proc. R. Soc. A. Mat. 471, 20150141 (10.1098/rspa.2015.0141)

[33] Holloway C, Smith D, Dyson R 2018 Linear Rayleigh–Benard stability of a transversely isotropic fluid. Eur. J. Appl. Math. 30, 1–23. (10.1017/s0956792518000359)

[34] Green J, Friedman A 2008 The extensional flow of a thin sheet of incompressible, transversely isotropic fluid. Eur. J. Appl. Math. 19, 225–257. (10.1017/S0956792508007377)

[35] Dyson R, Jensen O 2010 A fibre-reinforced fluid model of anisotropic plant cell growth. J. Fluid Mech. 655, 472–503. (10.1017/S002211201000100X)

[36] Cupples G, Dyson R, Smith D 2017 Viscous propulsion in active transversely isotropic media. J. Fluid Mech. 812, 501–524. (10.1017/jfm.2016.821)

[37] Cupples G, Dyson R, Smith D 2018 On viscous propulsion in active transversely isotropic media. J. Fluid Mech. 855, 408–420. (10.1017/jfm.2018.647)

[38] Dyson R, Green J, Whiteley J, Byrne H 2016 An investigation of the influence of extracellular matrix anisotropy and cell–matrix interactions on tissue architecture. J. Math. Biol. 72, 1775–1809. (10.1007/s00285-015-0927-7) 26328534

[39] Doi M, Edwards S 1978 Dynamics of rod–like macromolecules in concentrated solution. Part 1. J. Chem. Soc. Farad. Trans. 2 74, 560–570. (10.1039/f29787400560)

[40] Hinch E, Leal L 1975 Constitutive equations in suspension mechanics. Part 1. General formulation. J. Fluid Mech. 71, 481–495. (10.1017/S0022112075002698)

[41] Hinch E, Leal L 1976 Constitutive equations in suspension mechanics. Part 2. Approximate forms for a suspension of rigid particles affected by Brownian rotations. J. Fluid Mech. 76, 187–208. (10.1017/S0022112076003200)

[42] Nitsche L, Hinch E 1997 Shear-induced lateral migration of Brownian rigid rods in parabolic channel flow. J. Fluid Mech. 332, 1–21. (10.1017/S0022112096003369)

[43] Holloway C, Cupples G, Smith D, Green J, Clarke R, Dyson R 2018 Influences of transversely isotropic rheology and translational diffusion on the stability of active suspensions. R. Soc. open sci. 5, 180456 (10.1098/rsos.180456) 30225034 PMC6124136

[44] Garab G, van Amerongen H 2009 Linear dichroism and circular dichroism in photosynthesis research. Photosyn. Res. 101, 135–146. (10.1007/s11120-009-9424-4) 19418239 PMC2744782

[45] Bird R, Armstrong R, Hassager O, Curtiss C 1977 Dynamics of polymeric liquids, vol. 1 New York, NY: Wiley.

[46] Douglas J, Peaceman D 1955 Numerical solution of two-dimensional heat-flow problems. AIChE J. 1, 505–512. (10.1002/aic.690010421)

[47] Aubrey K, Thomas G Jr 1991 Raman spectroscopy of filamentous bacteriophage Ff (fd, M13, f1) incorporating specifically-deuterated alanine and tryptophan side chains. Assignments and structural interpretation. Biophys. J. 60, 1337–1349. (10.1016/S0006-3495(91)82171-3) 1777561 PMC1260194

[48] Glucksman M, Bhattacharjee S, Makowski L 1992 Three-dimensional structure of a cloning vector: X-ray diffraction studies of filamentous bacteriophage M13 at 7 Å resolution. J. Mol. Biol. 226, 455–470. (10.1016/0022-2836(92)90960-R) 1640460

[49] Niu Z, Bruckman M, Harp B, Mello C, Wang Q 2008 Bacteriophage M13 as a scaffold for preparing conductive polymeric composite fibers. Nano Res. 1, 235–241. (10.1007/s12274-008-8027-2)

[50] Pozrikidis C 2011 Introduction to theoretical and computational fluid dynamics. Oxford, UK: Oxford University Press.

[51] Khalil A, Ferrer J, Brau R, Kottmann S, Noren C, Lang M, Belcher A 2007 Single M13 bacteriophage tethering and stretching. Proc. Natl Acad. Sci. USA 104, 4892–4897. (10.1073/pnas.0605727104) 17360403 PMC1829235

[52] Hwang Y, Pedley T 2014 Bioconvection under uniform shear: linear stability analysis. J. Fluid Mech. 738, 522–562. (10.1017/jfm.2013.604)

[53] Koch D, Subramanian G 2011 Collective hydrodynamics of swimming microorganisms: living fluids. Annu. Rev. Fluid Mech. 43, 637–659. (10.1146/annurev-fluid-121108-145434)

[54] Saintillan D, Shelley M 2008 Instabilities, pattern formation, and mixing in active suspensions. Phys. Fluids 20, 123304 (10.1063/1.3041776) 18518342

[55] Subramanian G, Koch D, Fitzgibbon S 2011 The stability of a homogeneous suspension of chemotactic bacteria. Phys. Fluids 23, 041901 (10.1063/1.3580271)

[56] López H, Gachelin J, Douarche C, Auradou H, Clément E 2015 Turning bacteria suspensions into superfluids. Phys. Rev. Lett. 115, 028301 (10.1103/PhysRevLett.115.028301) 26207507

[57] Bearon R, Hazel A 2015 The trapping in high-shear regions of slender bacteria undergoing chemotaxis in a channel. J. Fluid Mech. 771, 2015198 (10.1017/jfm.2015.198)

