# Supplementary material for: Oriented suspension mechanics with application to improving flow linear dichroism spectroscopy
Source: Proc Math Phys Eng Sci. 2019 Dec 18;475(2232):20190184. doi: 10.1098/rspa.2019.0184 (PMC6936618; doi:10.1098/rspa.2019.0184)
Supplement: Summary of numerical methods [file rspa20190184supp1.pdf]

Oriented suspension mechanics with applications to improving flow linear dichroism spectroscopy: Supplemental material

G. Cupples, D. J. Smith, M. R. Hicks and R. J. Dyson

S1 Description of extra stress parameters

Each parameter (α_i, $i = 2, \ldots, 5$, α_r) associated with the extra stress terms (2.11) and (2.12) can be represented in terms of ellipsoidal integrals as

$$\begin{gathered}\alpha_2 = \frac{1}{I_1}\left(1 + \frac{L_1}{L_2} - 2\frac{I_1}{I_2}\right), \quad \alpha_3 = -\frac{1}{I_1}\left(1 - \frac{I_1}{I_2}\right), \quad \alpha_4 = \frac{1}{I_1}, \\ \alpha_5 = \frac{1}{3I_1}\left(1 - \frac{L_1}{L_2}\right), \quad \alpha_r = \frac{6(r^4-1)}{2r^2 I_2 + (r^2+1)^2 L_2}.\end{gathered} \tag{S1}$$

The integrals I_1, I_2, L_1 and L_2 are given in terms of the aspect ratio r by

$$I_1 = \int_0^\infty \frac{2r}{(r^2+\lambda)^{1/2}(1+\lambda)^3}\,\mathrm{d}\lambda = \frac{r^2(2r^2 - 5 + 3\gamma)}{2(r^2-1)^2}, \tag{S2}$$

$$I_2 = \int_0^\infty \frac{r(r^2+1)}{(r^2+\lambda)^{3/2}(1+\lambda)^2}\,\mathrm{d}\lambda = \frac{(r^2+1)(r^2+2-3r^2\gamma)}{2(r^2-1)^2}, \tag{S3}$$

$$L_1 = \int_0^\infty \frac{r\lambda}{(r^2+\lambda)^{3/2}(1+\lambda)^3}\,\mathrm{d}\lambda = \frac{r^2\left[2r^2+1-\gamma(4r^2-1)\right]}{4(r^2-2)^2}, \tag{S4}$$

$$L_2 = \int_0^\infty \frac{r\lambda}{(r^2+\lambda)^{3/2}(1+\lambda)^2}\,\mathrm{d}\lambda = I_1 - 2L_1, \tag{S5}$$

where

$$\gamma = \frac{\cosh^{-1} r}{r(r^2-1)^{1/2}}.$$

S2 Constants for the solution of the Fokker-Planck equation

The seven non-zero constants in equations (3.7) and (3.8) are

$$a_{n,n-2}^{m,m-2} = \frac{(n-2)(n+m)!(1-\delta_{m0})}{4(2n+1)(2n-1)(n+m-4)!}, \tag{S6}$$

$$a_{n,n}^{m,m-2} = \frac{3(n-m+2)!(n+m)!(1-\delta_{m0})}{4(2n+3)(2n-1)(n+m-2)!(n-m)!}, \tag{S7}$$

$$a_{n,n+2}^{m,m-2} = -\frac{(n+3)(n-m+4)!(1-\delta_{m0})}{4(2n+1)(2n+3)(n-m)!}, \tag{S8}$$

$$a_{n,n}^{m,m} = -\frac{m}{2}, \tag{S9}$$

$$a_{n,n-2}^{m,m+2} = -\frac{(n-2)(1+\delta_{m0})}{4(2n+1)(2n-1)}, \tag{S10}$$

$$a_{n,n}^{m,m+2} = -\frac{3(1+\delta_{m0})}{4(2n+3)(2n-1)}, \tag{S11}$$

$$a_{n,n+2}^{m,m+2} = \frac{(n+3)(1+\delta_{m0})}{4(2n+1)(2n+3)}. \tag{S12}$$

S3 Numerical solution for steady flow of an elongated particle suspension

The steady flow problem is solved using a finite difference scheme and constructing a matrix equation which is solved numerically. Due to the similarities in the method, the Newtonian equation is discussed in detail and the suspension problem briefly afterwards.

The Newtonian flow equations are

$$-1 = \frac{\partial^2 u}{\partial y^2} + \frac{\partial^2 u}{\partial z^2}\,, \tag{S13}$$

which is discretised as

$$-1 = -2\left(\frac{1}{\Delta y^2} + \frac{1}{\Delta z^2}\right) u_{i,j} + \frac{1}{\Delta y^2}\left(u_{i+1,j} + u_{i-1,j}\right) + \frac{1}{\Delta z^2}\left(u_{i,j+1} + u_{i,j-1}\right), \tag{S14}$$

for step sizes Δy and Δz and where $i = 1, 2, \dots, Y$ and $j = 1, 2, \dots Z$ represent gridpoints in y and z respectively.

Express equation (S14) in a matrix system $\mathbf{A}\boldsymbol{u} = \boldsymbol{b}$, where $\boldsymbol{u}$ is the vector

$$\boldsymbol{u} = (u_{1,1},\, u_{1,2},\, \dots u_{1,Z},\, u_{2,1}, \dots\, u_{i,j},\, \dots\, u_{Y,1},\, \dots u_{Y,Z})^T, \tag{S15}$$

and by constructing a sparse $YZ \times YZ$ matrix,

$$\mathbf{A} = \begin{pmatrix} \mathbf{I} & \mathbf{0} & \mathbf{0} & \cdots & \cdots & \cdots & \mathbf{0} \\ \mathbf{B} & \mathbf{C} & \mathbf{B} & \mathbf{0} & \cdots & \cdots & \mathbf{0} \\ \mathbf{0} & \mathbf{B} & \mathbf{C} & \mathbf{B} & \mathbf{0} & & \vdots \\ \vdots & \ddots & \ddots & \ddots & \ddots & \ddots & \vdots \\ \vdots & & \ddots & \ddots & \ddots & \ddots & \mathbf{0} \\ \mathbf{0} & \cdots & \cdots & \mathbf{0} & \mathbf{B} & \mathbf{C} & \mathbf{B} \\ \mathbf{0} & \cdots & \cdots & \cdots & \mathbf{0} & \mathbf{0} & \mathbf{I} \end{pmatrix}. \tag{S16}$$

Here, $\mathbf{B}$, $\mathbf{C}$, $\mathbf{I}$ and $\mathbf{0}$ are $Z \times Z$ matrices arranged in the $Y \times Y$ matrix (S16); $\mathbf{I}$ is the identity matrix, $\mathbf{0}$ is a matrix of zeros and

$$\mathbf{B} = \begin{pmatrix} 1 & 0 & 0 & \dots & \dots & 0 \\ 0 & r_y & 0 & \dots & \dots & 0 \\ \vdots & \ddots & \ddots & \ddots & & \vdots \\ \vdots & & \ddots & \ddots & \ddots & \vdots \\ 0 & & & 0 & r_y & 0 \\ 0 & \dots & \dots & 0 & 0 & 1 \end{pmatrix}, \qquad \mathbf{C} = \begin{pmatrix} 1 & 0 & 0 & \dots & \dots & 0 \\ r_z & -2r & r_z & 0 & \dots & 0 \\ 0 & r_z & -2r & r_z & \ddots & \vdots \\ \vdots & \ddots & \ddots & \ddots & \ddots & 0 \\ 0 & \dots & 0 & r_z & -2r & r_z \\ 0 & \dots & \dots & 0 & 0 & 1 \end{pmatrix}, \tag{S17}$$

where $r_y = 1/\Delta y^2$, $r_z = 1/\Delta z^2$ and $r = \left(1/\Delta y^2 + 1/\Delta z^2\right)$. The right hand side vector consists of minus one entries, where the corresponding no-slip boundary conditions are set to zero, *i.e.* entries $i = 1, Y$ and $j = 1, Z$. This system is solved using the direct solver backslash in Matlab and the resulting vector is reshaped to obtain a $Y \times Z$ matrix for the velocity u. From here, the velocity is re-dimensionalised and the shear rate can be calculated at each point in y^* and z^* as

$$\dot{\gamma}^* = \sqrt{\left(\frac{\partial u^*}{\partial y^*}\right)^2 + \left(\frac{\partial u^*}{\partial z^*}\right)^2}. \tag{S18}$$

The suspension model, given by equation (3.11) is rewritten as,

$$\begin{aligned} -1 - F_{i,j} = &\begin{cases} G_{i,j}\dfrac{u_{i,j} - u_{i-1,j}}{\Delta y} & \text{for } G_{i,j} \geq 0 \\ G_{i,j}\dfrac{u_{i+1,j} - u_{i,j}}{\Delta y} & \text{for } G_{i,j} < 0 \end{cases} + \begin{cases} H_{i,j}\dfrac{u_{i,j} - u_{i,j-1}}{\Delta z} & \text{for } H_{i,j} \geq 0 \\ H_{i,j}\dfrac{u_{i,j+1} - u_{i,j}}{\Delta z} & \text{for } H_{i,j} < 0 \end{cases} \\ &+ J_{i,j}\frac{u_{i+1,j} - 2u_{i,j} + u_{i-1,j}}{\Delta y^2} + K_{i,j}\frac{u_{i,j+1} - 2u_{i,j} + u_{i,j-1}}{\Delta z^2} \\ &+ L_{i,j}\frac{u_{i+1,j+1} - u_{i-1,j+1} - u_{i+1,j-1} + u_{i-1,j-1}}{4\Delta y \Delta z}, \end{aligned} \tag{S19}$$

where the second order derivatives are discretised via second order central differences, an upwind scheme has been used to deal with first order derivatives and

$$\mathbf{F} = \frac{2\Phi\alpha_r}{P_G}\left(\frac{\partial}{\partial y}\int_{\boldsymbol{s}} p_1 p_2 \psi \; \mathrm{d}\boldsymbol{p} + \frac{\partial}{\partial z}\int_{\boldsymbol{s}} p_1 p_3 \psi \; \mathrm{d}\boldsymbol{p}\right), \tag{S20}$$

$$\begin{aligned} \mathbf{G} = &4\Phi\frac{\partial}{\partial y}\left\{\alpha_2\int_{\boldsymbol{s}} p_1^2 p_2^2 \psi \; \mathrm{d}\boldsymbol{p} + \frac{\alpha_3}{2}\int_{\boldsymbol{s}} (p_1^2 + p_2^2)\psi \; \mathrm{d}\boldsymbol{p}\right\} \\ &+ 4\Phi\frac{\partial}{\partial z}\left\{\alpha_2\int_{\boldsymbol{s}} p_1^2 p_2 p_3 \psi \; \mathrm{d}\boldsymbol{p} + \frac{\alpha_3}{2}\int_{\boldsymbol{s}} p_2 p_3 \psi \; \mathrm{d}\boldsymbol{p}\right\}, \end{aligned} \tag{S21}$$

$$\begin{aligned} \mathbf{H} = &4\Phi\frac{\partial}{\partial z}\left\{\alpha_2\int_{\boldsymbol{s}} p_1^2 p_3^2 \psi \; \mathrm{d}\boldsymbol{p} + \frac{\alpha_3}{2}\int_{\boldsymbol{s}} (p_1^2 + p_3^2)\psi \; \mathrm{d}\boldsymbol{p}\right\} \\ &+ 4\Phi\frac{\partial}{\partial y}\left\{\alpha_2\int_{\boldsymbol{s}} p_1^2 p_2 p_3 \psi \; \mathrm{d}\boldsymbol{p} + \frac{\alpha_3}{2}\int_{\boldsymbol{s}} p_2 p_3 \psi \; \mathrm{d}\boldsymbol{p}\right\}, \end{aligned} \tag{S22}$$

$$\mathbf{J} = 1 + 4\Phi\left[\alpha_2\int_{\boldsymbol{s}} p_1^2 p_2^2 \psi \; \mathrm{d}\boldsymbol{p} + \frac{\alpha_3}{2}\int_{\boldsymbol{s}} (p_1^2 + p_2^2)\psi \; \mathrm{d}\boldsymbol{p} + \frac{\alpha_4}{2}\right], \tag{S23}$$

$$\mathbf{K} = 1 + 4\Phi\left[\alpha_2\int_{\boldsymbol{s}} p_1^2 p_3^2 \psi \; \mathrm{d}\boldsymbol{p} + \frac{\alpha_3}{2}\int_{\boldsymbol{s}} (p_1^2 + p_3^2)\psi \; \mathrm{d}\boldsymbol{p} + \frac{\alpha_4}{2}\right], \tag{S24}$$

$$\mathbf{L} = 8\Phi\left\{\alpha_2\int_{\boldsymbol{s}} p_1^2 p_2 p_3 \psi \; \mathrm{d}\boldsymbol{p} + \frac{\alpha_3}{2}\int_{\boldsymbol{s}} p_2 p_3 \psi \; \mathrm{d}\boldsymbol{p}\right\}. \tag{S25}$$

The operator matrix $\mathbf{A}$ takes on a similar form to (S16), and the right hand side vector is now

given by $\boldsymbol{b} = -\mathbf{1} - \mathbf{F}$, where $\mathbf{F}$ is restructured in the same way as the velocity. The matrix $\mathbf{A}$ is

$$\mathbf{A} = \begin{pmatrix} \mathbf{I} & \mathbf{0} & \mathbf{0} & \cdots & \cdots & \cdots & \mathbf{0} \\ \mathbf{B}_2 & \mathbf{C}_2 & \mathbf{D}_2 & \mathbf{0} & \cdots & \cdots & \mathbf{0} \\ \mathbf{0} & \mathbf{B}_3 & \mathbf{C}_3 & \mathbf{D}_3 & \mathbf{0} & & \vdots \\ \vdots & \ddots & \ddots & \ddots & \ddots & \ddots & \vdots \\ \vdots & & \ddots & \ddots & \ddots & \ddots & \mathbf{0} \\ \mathbf{0} & \cdots & \cdots & \mathbf{0} & \mathbf{B}_{Y-1} & \mathbf{C}_{Y-1} & \mathbf{D}_{Y-1} \\ \mathbf{0} & \cdots & \cdots & \cdots & \mathbf{0} & \mathbf{0} & \mathbf{I} \end{pmatrix}. \tag{S26}$$

where the matrices $\mathbf{B}_i$, $\mathbf{C}_i$ and $\mathbf{D}_i$ change with each step in y and have the form:

$$\mathbf{B}_i = \begin{pmatrix} 0 & 0 & 0 & \cdots & \cdots & \cdots & 0 \\ E_{i,2} & C_{i,2}^{(w,0)} & -E_{i,2} & 0 & \cdots & \cdots & 0 \\ 0 & E_{i,3} & C_{i,3}^{(w,0)} & -E_{i,3} & 0 & & \vdots \\ \vdots & \ddots & \ddots & \ddots & \ddots & \ddots & \vdots \\ \vdots & & \ddots & \ddots & \ddots & \ddots & 0 \\ 0 & \cdots & \cdots & 0 & E_{i,Z-1} & C_{i,Z-1}^{(w,0)} & -E_{i,Z-1} \\ 0 & \cdots & \cdots & \cdots & 0 & 0 & 0 \end{pmatrix}, \tag{S27}$$

$$\mathbf{C}_i = \begin{pmatrix} 1 & 0 & 0 & \cdots & \cdots & \cdots & 0 \\ C_{i,2}^{(0,w)} & C_{i,2}^{(0,0)} & C_{i,2}^{(0,e)} & 0 & \cdots & \cdots & 0 \\ 0 & C_{i,3}^{(0,w)} & C_{i,3}^{(0,0)} & C_{i,3}^{(0,e)} & 0 & & \vdots \\ \vdots & \ddots & \ddots & \ddots & \ddots & \ddots & \vdots \\ \vdots & & \ddots & \ddots & \ddots & \ddots & 0 \\ 0 & \cdots & \cdots & 0 & C_{i,Z-1}^{(0,w)} & C_{i,Z-1}^{(0,0)} & C_{i,Z-1}^{(0,e)} \\ 0 & \cdots & \cdots & \cdots & 0 & 0 & 1 \end{pmatrix}, \tag{S28}$$

and

$$\mathbf{D}_i = \begin{pmatrix} 0 & 0 & 0 & \cdots & \cdots & \cdots & 0 \\ -E_{i,2} & C_{i,2}^{(e,0)} & E_{i,2} & 0 & \cdots & \cdots & 0 \\ 0 & -E_{i,3} & C_{i,3}^{(e,0)} & E_{i,3} & 0 & & \vdots \\ \vdots & \ddots & \ddots & \ddots & \ddots & \ddots & \vdots \\ \vdots & & \ddots & \ddots & \ddots & \ddots & 0 \\ 0 & \cdots & \cdots & 0 & -E_{i,Z-1} & C_{i,Z-1}^{(e,0)} & E_{i,Z-1} \\ 0 & \cdots & \cdots & \cdots & 0 & 0 & 0 \end{pmatrix}. \tag{S29}$$

The various matrix entries are given by expressions relating to (S20)–(S25) and depend upon the sign of $\mathbf{G}$ and $\mathbf{H}$ at each point in space. They are summarised for each case as follows:

For $G_{i,j}$, $H_{i,j} \geq 0$,

$$C_{i,j}^{(0,0)} = -2\left(\frac{J_{i,j}}{\Delta y^2} + \frac{K_{i,j}}{\Delta z^2}\right) + \frac{G_{i,j}}{\Delta y} + \frac{H_{i,j}}{\Delta z}, \qquad C_{i,j}^{(e,0)} = \frac{J_{i,j}}{\Delta y^2},$$
$$C_{i,j}^{(w,0)} = \frac{J_{i,j}}{\Delta y^2} - \frac{G_{i,j}}{\Delta y}, \qquad C_{i,j}^{(0,e)} = \frac{K_{i,j}}{\Delta z^2}, \qquad C_{i,j}^{(0,w)} = \frac{K_{i,j}}{\Delta z^2} - \frac{H_{i,j}}{\Delta z}. \tag{S30}$$

For $G_{i,j} \geq 0$ and $H_{i,j} < 0$,

$$C_{i,j}^{(0,0)} = -2\left(\frac{J_{i,j}}{\Delta y^2} + \frac{K_{i,j}}{\Delta z^2}\right) + \frac{G_{i,j}}{\Delta y} - \frac{H_{i,j}}{\Delta z}, \qquad C_{i,j}^{(e,0)} = \frac{J_{i,j}}{\Delta y^2},$$
$$C_{i,j}^{(w,0)} = \frac{J_{i,j}}{\Delta y^2} - \frac{G_{i,j}}{\Delta y}, \qquad C_{i,j}^{(0,e)} = \frac{K_{i,j}}{\Delta z^2} + \frac{H_{i,j}}{\Delta z}, \qquad C_{i,j}^{(0,w)} = \frac{K_{i,j}}{\Delta z^2}. \tag{S31}$$

For $G_{i,j} < 0$ and $H_{i,j} \geq 0$,

$$C_{i,j}^{(0,0)} = -2\left(\frac{J_{i,j}}{\Delta y^2} + \frac{K_{i,j}}{\Delta z^2}\right) - \frac{G_{i,j}}{\Delta y} + \frac{H_{i,j}}{\Delta z}, \qquad C_{i,j}^{(e,0)} = \frac{J_{i,j}}{\Delta y^2} + \frac{G_{i,j}}{\Delta y},$$
$$C_{i,j}^{(w,0)} = \frac{J_{i,j}}{\Delta y^2}, \qquad C_{i,j}^{(0,e)} = \frac{K_{i,j}}{\Delta z^2}, \qquad C_{i,j}^{(0,w)} = \frac{K_{i,j}}{\Delta z^2} - \frac{H_{i,j}}{\Delta z}. \tag{S32}$$

For $G_{i,j}$, $H_{i,j} < 0$,

$$C_{i,j}^{(0,0)} = -2\left(\frac{J_{i,j}}{\Delta y^2} + \frac{K_{i,j}}{\Delta z^2}\right) - \frac{G_{i,j}}{\Delta y} - \frac{H_{i,j}}{\Delta z}, \qquad C_{i,j}^{(e,0)} = \frac{J_{i,j}}{\Delta y^2} + \frac{G_{i,j}}{\Delta y},$$
$$C_{i,j}^{(w,0)} = \frac{J_{i,j}}{\Delta y^2}, \qquad C_{i,j}^{(0,e)} = \frac{K_{i,j}}{\Delta z^2} + \frac{H_{i,j}}{\Delta z}, \qquad C_{i,j}^{(0,w)} = \frac{K_{i,j}}{\Delta z^2}. \tag{S33}$$

A direct solver (backslash) in Matlab is used to evaluate this matrix equation and the solution is reconstructed to create a matrix for $\boldsymbol{u}$ in y and z.

The convergence of the probability density function ψ and velocity u^* is checked by varying the number of modes in the spherical harmonic discretisation and grid size for the finite difference approximation respectively.

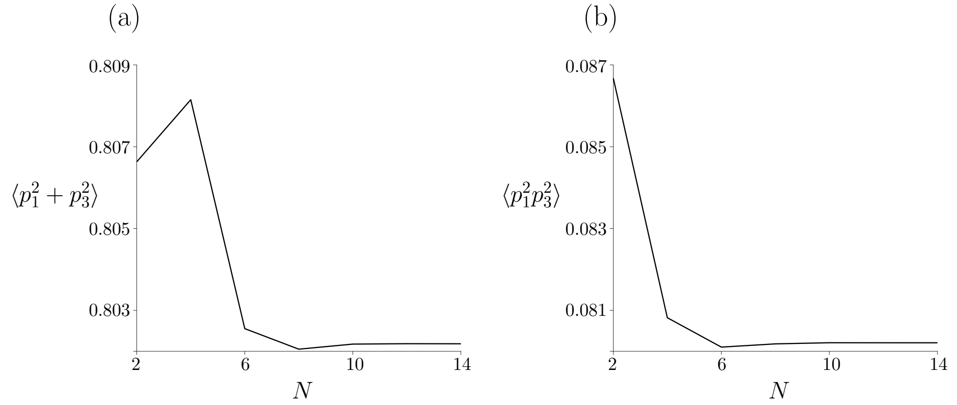

Figure S1: Convergence of spatially averaged orientation tensors for steady flow: (a) $\langle p_1^2 + p_3^2 \rangle$ and (b) $\langle p_1^2 p_3^2 \rangle$ for varying number of modes N. Convergence is achieved for $N = 10$.

The accuracy of the spherical harmonic discretisation is evaluated by considering moments of ψ,

$$\langle p_1^2 + p_3^2 \rangle = \int_{-\pi}^{\pi} \int_0^{\pi} p_1^2 + p_3^2 \, \psi \sin\theta \, \mathrm{d}\theta \, \mathrm{d}\phi$$
$$= \int_{-\pi}^{\pi} \int_0^{\pi} (\sin^2\theta \cos^2\phi + \cos^2\theta) \, \psi \sin\theta \, \mathrm{d}\theta \, \mathrm{d}\phi, \tag{S34}$$

$$\langle p_1^2 p_3^2 \rangle = \int_{-\pi}^{\pi} \int_0^{\pi} p_1^2 p_3^2 \, \psi \sin\theta \, \mathrm{d}\theta \, \mathrm{d}\phi$$
$$= \int_{-\pi}^{\pi} \int_0^{\pi} \sin^3\theta \cos^2\theta \cos^2\phi \, \psi \, \mathrm{d}\theta \, \mathrm{d}\phi. \tag{S35}$$

Figure S1 (a) and (b) show $\langle p_1^2 + p_3^2 \rangle$ and $\langle p_1^2 p_3^2 \rangle$ respectively, averaged over the channel, with increasing N. Once $N = 10$ the value of these moments do not change, hence this is our choice for all numerical calculations.

Next the convergence of the finite difference approximation for the number of steps Y and Z is tested by comparing the spatially averaged velocity u^* (figure S2). The results agree to within 0.01% at $Y = 150$ and $Z = 120$.

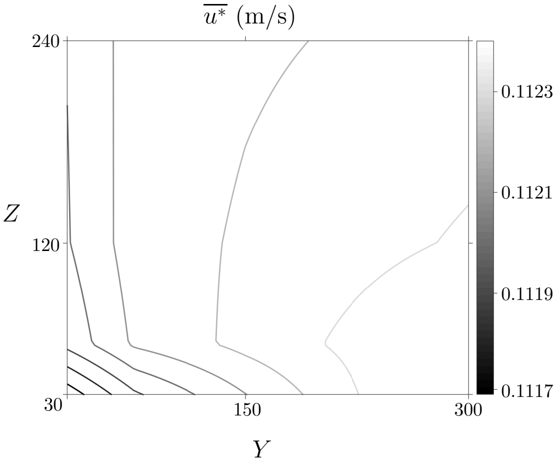

Figure S2: Convergence of the spatially averaged steady flow velocity $\overline{u^*}$ for an increasing number of steps in y^* and z^*. The grid points where convergence is achieved are $Y = 150$ and $Z = 120$.

S4 Iterative solution for the coupled steady flow model

The coupled model consists of equations (3.9)–(3.12); the flow and orientation distribution equations are numerically coupled using an iterative approach. Initially the Newtonian problem is solved and the shear rate,

$$\dot{\gamma}^* = \sqrt{\left(\frac{\partial u^*}{\partial y^*}\right)^2 + \left(\frac{\partial u^*}{\partial z^*}\right)^2},$$

is calculated. At each point in space, the angles θ and ϕ are rotated into a local coordinate system, determined by the velocity gradients at that point. Then the orientation distribution function, and hence the fluid flow equations, are updated.

A summary of the iterative process is as follows:

- Calculate velocity profile, $u^{(j)}(y,z)$, from equation (3.11), where the Newtonian profile is used when $j=0$, and re-dimensionalise.
- Evaluate the probability density function $\psi^{(j)}(\phi,\theta,y^*,z^*)$ by solving the system (3.9)–(3.10) and via the discretisation (3.4).
- Calculate the moments of $\psi^{(j)}(\phi,\theta,y^*,z^*)$, and hence update the velocity $u^{(j+1)}(y,z)$ in equation (3.11).
- The dimensional velocity $u^{*(j+1)}(y^*,z^*)$ can then be used to calculate the probability density function $\psi^{(j+1)}(\phi,\theta,\phi,y^*,z^*)$ from (3.9)–(3.10) and (3.4).
- Repeat the above steps until the iteration terminates, *i.e.* when

$$||u^{(j+1)} - u^{(j)}|| < \tau,$$

  for some tolerance τ. We require an absolute tolerance of $\tau = 10^{-6}$ in our numerical calculation.

S5 Numerical solution to the oscillatory flow problem for an elongated particle suspension

The oscillating flow equations are solved by employing an ADI method and constructing matrix equations, that are solved numerically. For a Newtonian fluid we have,

$$\alpha^2\frac{\partial u}{\partial t} = -\exp\left(i\left[t+\frac{\pi}{2}\right]\right) + \frac{\partial^2 u}{\partial y^2} + \frac{\partial^2 u}{\partial z^2}\,, \tag{S36}$$

discretise and split the time step in half to get

$$\begin{aligned}\alpha^2\frac{u_{i,j}^{n+1/2} - u_{i,j}^{n}}{\Delta t/2} &= -\exp\left(i\left[t^{n+1/2}+\frac{\pi}{2}\right]\right) + \frac{u_{i+1,j}^{n+1/2} - 2u_{i,j}^{n+1/2} + u_{i-1,j}^{n+1/2}}{\Delta y^2}\\ &\quad + \frac{u_{i,j+1}^{n} - 2u_{i,j}^{n} + u_{i,j-1}^{n}}{\Delta z^2},\end{aligned} \tag{S37}$$

$$\begin{aligned}\alpha^2\frac{u_{i,j}^{n+1} - u_{i,j}^{n+1/2}}{\Delta t/2} &= -\exp\left(i\left[t^{n+1}+\frac{\pi}{2}\right]\right) + \frac{u_{i+1,j}^{n+1/2} - 2u_{i,j}^{n+1/2} + u_{i-1,j}^{n+1/2}}{\Delta y^2}\\ &\quad + \frac{u_{i,j+1}^{n+1} - 2u_{i,j}^{n+1} + u_{i,j-1}^{n+1}}{\Delta z^2}.\end{aligned} \tag{S38}$$

The first half time step is implicit in y and is solved for each point in z, the second half time step uses the solution to the first equation, $u_{i,j}^{n+1/2}$, as the explicit velocity and is solved implicitly in z and explicitly in y. Rearranging each of the above,

$$\begin{aligned}\left(1+\frac{\Delta t}{\alpha^2\Delta y^2}\right)u_{i,j}^{n+1/2} - \frac{\Delta t}{2\alpha^2\Delta y^2}(u_{i+1,j}^{n+1/2} + u_{i-1,j}^{n+1/2}) &= \frac{\Delta t}{2\alpha^2\Delta z^2}(u_{i,j+1}^{n} + u_{i,j-1}^{n})\\ &\quad -\frac{\Delta t}{2\alpha^2}\exp\left(i\left[t^{n+1/2}+\frac{\pi}{2}\right]\right) + \left(1-\frac{\Delta t}{\alpha^2\Delta z^2}\right)u_{i,j}^{n},\end{aligned} \tag{S39}$$

$$\left(1+\frac{\Delta t}{\alpha^2\Delta z^2}\right)u_{i,j}^{n+1}-\frac{\Delta t}{2\alpha^2\Delta z^2}(u_{i,j+1}^{n+1}+u_{i,j-1}^{n+1})=\frac{\Delta t}{2\alpha^2\Delta y^2}(u_{i+1,j}^{n+1/2}+u_{i-1,j}^{n+1/2}) \\ -\frac{\Delta t}{2\alpha^2}\exp\left(i\left[t^{n+1}+\frac{\pi}{2}\right]\right)+\left(1-\frac{\Delta t}{\alpha^2\Delta y^2}\right)u_{i,j}^{n+1/2} \tag{S40}$$

which are both solved via a matrix equation $\mathbf{A}\boldsymbol{u}=\boldsymbol{b}$. The operator matrix for the first time step is

$$\mathbf{A}=\begin{pmatrix} 1 & 0 & 0 & \dots & \dots & \dots & 0 \\ C_w & C_0 & C_e & 0 & \dots & \dots & 0 \\ 0 & C_w & C_0 & C_e & 0 & & \vdots \\ \vdots & \ddots & \ddots & \ddots & \ddots & \ddots & \vdots \\ \vdots & & \ddots & \ddots & \ddots & \ddots & 0 \\ 0 & \dots & \dots & 0 & C_w & C_0 & C_e \\ 0 & \dots & \dots & \dots & 0 & 0 & 1 \end{pmatrix}, \tag{S41}$$

where $C_w=C_e=-\Delta t/(2\alpha^2\Delta y^2)$ and $C_0=1+\Delta t/(\alpha^2\Delta y^2)$. The right hand side vector $\boldsymbol{b}$ is given by the right hand side of equation (S39), where the first and last entries are zero to satisfy the boundary conditions. This is solved using the direct solver backslash in Matlab for each $j=2,\dots,Z-1$, and the boundary conditions $u_{i,1}^{n+1/2}=u_{i,Z}^{n+1/2}=0$ are enforced afterwards.

The second half time step follows the same method. Since no-slip is imposed in both y and z, the operator matrices have the same form; the entries for the second time step are now given by $C_w=C_e=-\Delta t/(2\alpha^2\Delta z^2)$ and $C_0=1+\Delta t/(\alpha^2\Delta z^2)$ and the right hand side vector is given by the right hand side of equation (S40) with equivalent boundary conditions. This is solved for each $i=2,\dots,Y-1$ and both the above steps are completed to update the velocity at time step t^{n+1}.

The suspension model contains coefficients of $u(y,z)$ in terms of moments of the orientation distribution $\psi(\phi,\theta,y^*,z^*,t^*)$. The numerical method is similar to the Newtonian problem and summarised here. Discretising equation (4.6) and splitting into two half-time steps we have

$$\alpha^2\frac{u_{i,j}^{n+1/2}-u_{i,j}^n}{\Delta t/2}=\exp\left(i\left[t^{n+1/2}+\frac{\pi}{2}\right]\right)+\begin{cases} G_{i,j}^{n+1/2}\dfrac{u_{i,j}^{n+1/2}-u_{i-1,j}^{n+1/2}}{\Delta y} & \text{for } G_{i,j}^{n+1/2}\geq 0 \\ G_{i,j}^{n+1/2}\dfrac{u_{i+1,j}^{n+1/2}-u_{i,j}^{n+1/2}}{\Delta y} & \text{for } G_{i,j}^{n+1/2}<0 \end{cases} \\ +\begin{cases} H_{i,j}^n\dfrac{u_{i,j}^n-u_{i,j-1}^n}{\Delta z} & \text{for } H_{i,j}^n\geq 0 \\ H_{i,j}^n\dfrac{u_{i,j+1}^n-u_{i,j}^n}{\Delta z} & \text{for } H_{i,j}^n<0 \end{cases}+K_{i,j}^n\frac{u_{i,j+1}^n-2u_{i,j}^n+u_{i,j-1}^n}{\Delta z^2} \\ +F_{i,j}^{n+1/2}+J_{i,j}^{n+1/2}\frac{u_{i+1,j}^{n+1/2}-2u_{i,j}^{n+1/2}+u_{i-1,j}^{n+1/2}}{\Delta y^2} \\ +L_{i,j}^n\frac{u_{i+1,j+1}^n-u_{i-1,j+1}^n-u_{i+1,j-1}^n+u_{i-1,j-1}^n}{4\Delta y\Delta z}, \tag{S42}$$

$$\alpha^2 \frac{u_{i,j}^{n+1} - u_{i,j}^{n+1/2}}{\Delta t/2} = \exp\left(i\left[t^{n+1/2} + \frac{\pi}{2}\right]\right) + \begin{cases} G_{i,j}^{n+1/2}\, \dfrac{u_{i,j}^{n+1/2} - u_{i-1,j}^{n+1/2}}{\Delta y} & \text{for } G_{i,j}^{n+1/2} \geq 0 \\ G_{i,j}^{n+1/2}\, \dfrac{u_{i+1,j}^{n+1/2} - u_{i,j}^{n+1/2}}{\Delta y} & \text{for } G_{i,j}^{n+1/2} < 0 \end{cases}$$
$$+ \begin{cases} H_{i,j}^{n+1}\, \dfrac{u_{i,j}^{n+1} - u_{i,j-1}^{n+1}}{\Delta z} & \text{for } H_{i,j}^{n+1} \geq 0 \\ H_{i,j}^{n+1}\, \dfrac{u_{i,j+1}^{n+1} - u_{i,j}^{n+1}}{\Delta z} & \text{for } H_{i,j}^{n+1} < 0 \end{cases} + K_{i,j}^{n+1} \frac{u_{i,j+1}^{n+1} - 2u_{i,j}^{n+1} + u_{i,j-1}^{n+1}}{\Delta z^2} \tag{S43}$$
$$+ F_{i,j}^{n+1} + J_{i,j}^{n+1/2} \frac{u_{i+1,j}^{n+1/2} - 2u_{i,j}^{n+1/2} + u_{i-1,j}^{n+1/2}}{\Delta y^2}$$
$$+ L_{i,j}^{n+1/2} \frac{u_{i+1,j+1}^{n+1/2} - u_{i-1,j+1}^{n+1/2} - u_{i+1,j-1}^{n+1/2} + u_{i-1,j-1}^{n+1/2}}{4\Delta y \Delta z},$$

where the coefficients $\mathbf{F}-\mathbf{L}$ are known expressions given by (S20)–(S25), which are time dependent. The operator matrices and right hand side vectors at each half-time step are now dependent on the sign of $\mathbf{G}$ and $\mathbf{H}$; all other factors of the method are unchanged. The operator matrix is given by (S41) and the terms are dependent on the sign of $\mathbf{G}$ as follows:
For $G_{i,j}^{n+1/2} \geq 0$,

$$C_0 = 1 + \frac{\Delta t}{\alpha^2 \Delta y}\left(\frac{J_{i,j}^{n+1/2}}{\Delta y} - \frac{G_{i,j}^{n+1/2}}{2}\right), \quad C_e = -\frac{\Delta t J_{i,j}^{n+1/2}}{\alpha^2 \Delta y^2},$$
$$C_w = -\frac{\Delta t}{\alpha^2 \Delta y^2}\left(\frac{J_{i,j}^{n+1/2}}{\Delta y} - G_{i,j}^{n+1/2}\right). \tag{S44}$$

For $G_{i,j}^{n+1/2} < 0$,

$$C_0 = 1 + \frac{\Delta t}{\alpha^2 \Delta y}\left(\frac{J_{i,j}^{n+1/2}}{\Delta y} + \frac{G_{i,j}^{n+1/2}}{2}\right), \quad C_e = -\frac{\Delta t}{\alpha^2 \Delta y^2}\left(\frac{J_{i,j}^{n+1/2}}{\Delta y} + G_{i,j}^{n+1/2}\right),$$
$$C_w = -\frac{\Delta t J_{i,j}^{n+1/2}}{\alpha^2 \Delta y^2}. \tag{S45}$$

The right hand side vector is given by

$$b_i = b_0^n u_{i,j}^n + b_e^n u_{i,j+1}^n + b_w^n u_{i,j-1}^n + \frac{\Delta t}{2\alpha^2}\left(F_{i,j}^{n+1/2} - \exp(i(t^{n+1/2} + \pi/2))\right)$$
$$+ \frac{\Delta t L_{i,j}^n}{8\alpha^2 \Delta y \Delta z}(u_{i+1,j+1}^n - u_{i-1,j+1}^n - u_{i+1,j-1}^n + u_{i-1,j-1}^n), \tag{S46}$$

where for $H_{i,j}^n \geq 0$,

$$b_0 = 1 - \frac{\Delta t}{\alpha^2 \Delta z}\left(\frac{K_{i,j}^n}{\Delta z} - \frac{H_{i,j}^n}{2}\right), \; b_e = \frac{\Delta t K_{i,j}^n}{2\alpha^2 \Delta z^2}, \; b_w = \frac{\Delta t}{2\alpha^2 \Delta z}\left(\frac{K_{i,j}^n}{\Delta z} - H_{i,j}^n\right), \tag{S47}$$

and for $H_{i,j}^n < 0$,

$$b_0 = 1 - \frac{\Delta t}{\alpha^2 \Delta z}\left(\frac{K_{i,j}^n}{\Delta z} + \frac{H_{i,j}^n}{2}\right),\ b_e = \frac{\Delta t}{2\alpha^2 \Delta z^2}\left(\frac{K_{i,j}^n}{\Delta z} + H_{i,j}^n\right),\ b_w = \frac{\Delta t K_{i,j}^n}{2\alpha^2 \Delta z}. \tag{S48}$$

The second time step is constructed similarly; the coefficients of the operator matrix can be defined as follows: For $H_{i,j}^{n+1} \geq 0$,

$$\begin{gathered} C_0 = 1 + \frac{\Delta t}{\alpha^2 \Delta z}\left(\frac{K_{i,j}^{n+1}}{\Delta z} - \frac{H_{i,j}^{n+1}}{2}\right), \quad C_e = -\frac{\Delta t K_{i,j}^{n+1}}{\alpha^2 \Delta z^2}, \\ C_w = -\frac{\Delta t}{\alpha^2 \Delta z^2}\left(\frac{K_{i,j}^{n+1}}{\Delta z} - H_{i,j}^{n+1}\right). \end{gathered} \tag{S49}$$

For $H_{i,j}^{n+1} < 0$,

$$\begin{gathered} C_0 = 1 + \frac{\Delta t}{\alpha^2 \Delta z}\left(\frac{K_{i,j}^{n+1}}{\Delta y} + \frac{H_{i,j}^{n+1}}{2}\right), \quad C_e = -\frac{\Delta t}{\alpha^2 \Delta z^2}\left(\frac{K_{i,j}^{n+1}}{\Delta z} + H_{i,j}^{n+1}\right), \\ C_w = -\frac{\Delta t K_{i,j}^{n+1}}{\alpha^2 \Delta z^2}. \end{gathered} \tag{S50}$$

The right hand side vector is given by

$$\begin{aligned} b_j = {} & b_0^{n+1/2} u_{i,j}^{n+1/2} + b_e^{n+1/2} u_{i,j+1}^{n+1/2} + b_w^{n+1/2} u_{i,j-1}^{n+1/2} + \frac{\Delta t}{2\alpha^2}\left(F_{i,j}^{n+1} - \exp(i(t^{n+1} + \pi/2))\right) \\ & + \frac{\Delta t L_{i,j}^{n+1/2}}{8\alpha^2 \Delta y \Delta z}(u_{i+1,j+1}^{n+1/2} - u_{i-1,j+1}^{n+1/2} - u_{i+1,j-1}^{n+1/2} + u_{i-1,j-1}^{n+1/2}), \end{aligned} \tag{S51}$$

where for $G_{i,j} \geq 0$,

$$\begin{gathered} b_0 = 1 - \frac{\Delta t}{\alpha^2 \Delta y}\left(\frac{J_{i,j}^{n+1/2}}{\Delta y} - \frac{G_{i,j}^{n+1/2}}{2}\right), \quad b_e = \frac{\Delta t J_{i,j}^{n+1/2}}{2\alpha^2 \Delta y^2}, \\ b_w = \frac{\Delta t}{2\alpha^2 \Delta y}\left(\frac{J_{i,j}^{n+1/2}}{\Delta y} - G_{i,j}^{n+1/2}\right), \end{gathered} \tag{S52}$$

and for $G_{i,j} < 0$,

$$\begin{gathered} b_0 = 1 - \frac{\Delta t}{\alpha^2 \Delta y}\left(\frac{J_{i,j}^{n+1/2}}{\Delta y} + \frac{G_{i,j}^{n+1/2}}{2}\right), \quad b_e = \frac{\Delta t}{2\alpha^2 \Delta y^2}\left(\frac{J_{i,j}^{n+1/2}}{\Delta y} + G_{i,j}^{n+1/2}\right), \\ b_w = \frac{\Delta t J_{i,j}^{n+1/2}}{2\alpha^2 \Delta y}. \end{gathered} \tag{S53}$$

The convergence of these numerical methods is now considered. First the spatial convergence of the finite difference approximation is checked (figure S4 (a)); the results agree with those in appendix S3 (figure S2). Next the time steps T are increased (figure S4 (b)) where $Y = 150$ and $Z = 120$. The variation between $T = 1200$ and $T = 1600$ is $\approx 0.01\%$ of the value of $\overline{u^*}$ and so we take $T = 1200$ for all calculations.

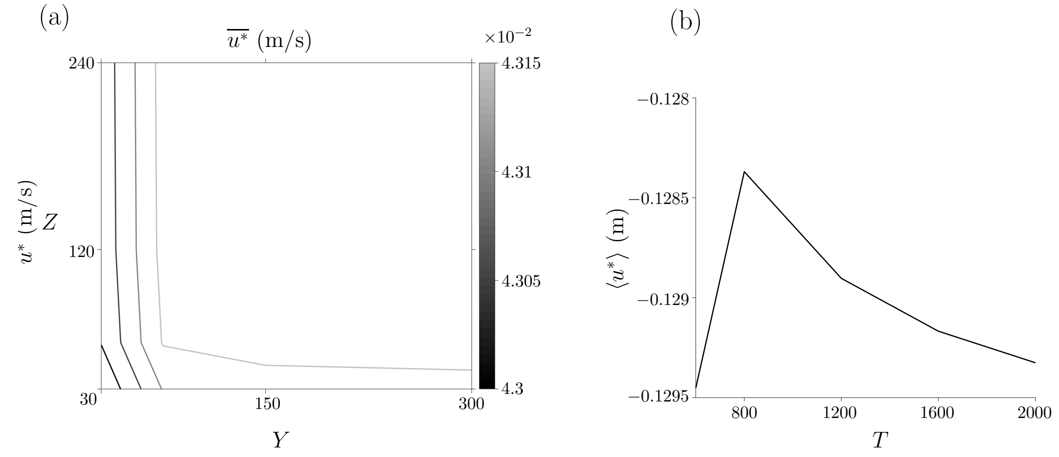

Figure S3: Convergence of the oscillating flow ADI scheme. (a) Gridpoints Y and Z for $T = 800$, where the convergence of the scheme is achieved for $Y = 150$ and $= 120$. (b) Time steps T for $Y = 120$ and $Z = 150$; convergence is achieved for $T = 1200$.

S6 Iterative solution for the coupled oscillatory flow model

As in section 3 and S4, the solution is numerically obtained via an iterative coupling of the orientation distribution and flow equations. Before initialising the iterative coupling, the associated Legendre polynomials $P_n^m(\cos\theta)$ for the orientation distribution function and all associated moments are calculated, for all possible rotation angles $\beta \in [0,\, 2\pi]$, and stored as a gridded interpolant. This step occurs only once throughout all calculations. Due to the numerical complexity, the iteration is performed within each time step and the process is modified as follows:

- Calculate the velocity profile over the first time step to obtain $u^{(j)}(y, z, t(2))$, where for $j = 0$ the Newtonian velocity is calculated.
- Re-dimensionalise the solution, then calculate the rotation angle at each point in space based on the shear rate.
- Solve the system (4.4)–(4.5) using an Improved Euler method over the same time step
- Evaluate the gridded interpolant for a given rotation angle β and reconstruct the probability density function $\psi^{(j)}(\phi, \theta, y^*, z^*, t^*(2))$, and its associated moments, using the discretisation (4.3).
- The updated velocity $u^{(j+1)}(y, z, t(2))$ is then calculated from the moments of $\psi^{(j)}(\phi, \theta, y^*, z^*, t^*(2))$ in equation (4.6).
- This iteration is repeated until

$$||u^{(j+1)}(y, z, t(2)) - u^{(j)}(y, z, t(2))|| < \tau,$$

  for some tolerance τ. We require an absolute tolerance of $\tau = 10^{-4}$ in our numerical calculation.
- Once this iteration converges, step through time and repeat the above process; the initial velocity $u^{(0)}(y, z, t(j+1))$ is first calculated using the moments from time step j.

S7 Calculation of parameter values

The calculation of a number of parameters in table 1 are explained here. The volume fraction can be written as $\Phi = V_c^* n_d^*$, where $V_c^* = 4\pi a^* b^{*2}/3$ is the volume of one particle and n_d^* is the particle number density. The bacteriophage in suspension have principal axis $a^* = 800$ nm and minor axes $b^* = c^* = 6$ nm, and hence volume $V_c^* \approx 1.21 \times 10^{-21}$ m^3. The number density of phage $n_d^* = C^* N_A/M^*$, with phage concentration $C^* = 0.02$ g/litre and molecular mass $M^* \approx 1.64 \times 10^7$ g/mol; the resulting value is $n_d^* \approx 7.33 \times 10^{17}$ phage/m^3, which provides a volume fraction $\Phi \approx 1.11 \times 10^{-5}$. Next, the global Péclet number $P_G = G^* h^*/D_r^* \mu^*$ is calculated. The dimensional diffusion coefficient D_r^* is

$$D_r^* = \frac{k_B^* T^*}{8\pi \mu^* a^* b^{*3} F_r}, \tag{S54}$$

where k_B^* is the Boltzmann constant, T^* is temperature and the rotational resistance coefficient is

$$F_r = \frac{4(r^4 - 1)}{3r^2 \left[\dfrac{2(2r^2 - 1)}{r^{3/4} F_t} - 2 \right]}, \tag{S55}$$

with

$$F_t = \frac{\sqrt{r^2 - 1}}{r^{1/3} \ln(r + \sqrt{r^2 - 1})}. \tag{S56}$$

Assuming the bacteriophage are suspended in water, we use the known viscosity at 22°C ($\approx$ 295K), $\mu^* = 9.5 \times 10^{-4}$ Pa s. Hence the diffusion coefficient is $D_r^* \approx 40.65$ s^{-1}. The dimensionless global Péclet number, calculated from the fixed pressure gradient, is $P_G \approx 34.17$; advection slightly dominates diffusion.

S8 Effect of number density on velocity and orientation parameter in steady flow

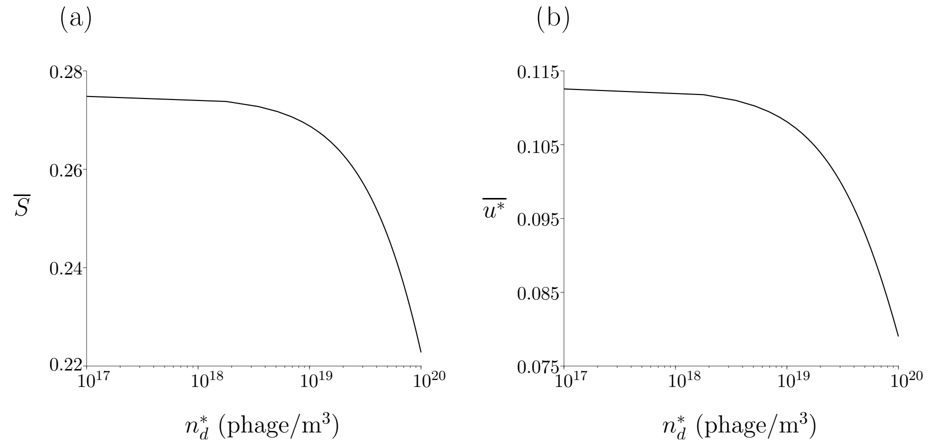

Figure S4: The spatially averaged orientation parameter and velocity with increasing number density. (a) Orientation parameter $\overline{S}$, (b) velocity $\overline{u^*}$.
